# Lack of impact of pre-existing T97A HIV-1 integrase mutation on integrase strand transfer inhibitor resistance and treatment outcome

**DOI:** 10.1371/journal.pone.0172206

**Published:** 2017-02-17

**Authors:** Michael E. Abram, Renee R. Ram, Nicolas A. Margot, Tiffany L. Barnes, Kirsten L. White, Christian Callebaut, Michael D. Miller

**Affiliations:** Gilead Sciences, Foster City, California, United States of America; Universidad Autonoma de Madrid Centro de Biologia Molecular Severo Ochoa, SPAIN

## Abstract

T97A is an HIV-1 integrase polymorphism associated with integrase strand transfer inhibitor (INSTI) resistance. Using pooled data from 16 clinical studies, we investigated the prevalence of T97A (pre-existing and emergent) and its impact on INSTI susceptibility and treatment response in INSTI-naive patients who enrolled on elvitegravir (EVG)- or raltegravir (RAL)-based regimens. Prior to INSTI-based therapy, primary INSTI resistance-associated mutations (RAMs) were absent and T97A pre-existed infrequently (1.4%; 47 of 3367 integrase sequences); most often among non-B (5.3%) than B (0.9%) HIV-1 subtypes. During INSTI-based therapy, few patients experienced virologic failure with emergent INSTI RAMs (3%; 122 of 3881 patients), among whom T97A emerged infrequently in the presence (n = 6) or absence (n = 8) of primary INSTI RAMs. A comparison between pre-existing and emergent T97A patient populations (i.e., in the absence of primary INSTI RAMs) showed no significant differences in EVG or RAL susceptibility in vitro. Furthermore, among all T97A-containing viruses tested, only 38–44% exhibited reduced susceptibility to EVG and/or RAL (all of low magnitude; <11-fold), while all maintained susceptibility to dolutegravir. Of the patients with pre-existing T97A, 17 had available clinical follow-up: 16 achieved virologic suppression and 1 maintained T97A and INSTI sensitivity without further resistance development. Overall, T97A is an infrequent integrase polymorphism that is enriched among non-B HIV-1 subtypes and can confer low-level reduced susceptibility to EVG and/or RAL. However, detection of T97A does not affect response to INSTI-based therapy with EVG or RAL. These results suggest a very low risk of initiating INSTI-based therapy in patients with pre-existing T97A.

## Introduction

Combination antiretroviral therapy has revolutionized HIV/AIDS management, reducing viral burden and HIV-related morbidity and mortality rates [1]. However, long-term efficacy of some of the earliest antiretroviral drug classes, including nucleos[t]ide reverse transcriptase inhibitors (NRTIs), non-nucleoside reverse transcriptase inhibitors (NNRTIs), and protease inhibitors (PIs), has been limited by the development of resistance-associated mutations (RAMs) at virologic failure [2]. The genetic variability and high evolution rate of HIV-1 support the hypothesis that some RAMs may naturally occur as minor variants before initiation of antiretroviral therapy [3–5], while others emerge as major variants under selective pressures during antiretroviral therapy [6]. Studies have shown that pre-existing minor NNRTI-resistant variants may be associated with poor treatment responses [7–11] and that transmitted drug resistance against NRTIs, NNRTIs, or PIs may delay virologic suppression and increase the risk of early virologic failure [12–15]. Consequently, genotypic testing for pre-existing drug resistance mutations in the protease and reverse transcriptase genes is recommended for the selection and modification of drug regimens [16, 17].

Integrase strand transfer inhibitors (INSTIs), including raltegravir (RAL; Isentress^®^) [18], elvitegravir (EVG; Vitekta^®^) [19], and dolutegravir (DTG; Tivicay^®^) [20], represent the newest class of approved antiretroviral drugs with demonstrated efficacy and safety in treatment-naive (TN) patients and heavily treatment-experienced (TE) patients with resistance to other antiretrovirals [21–32]. INSTI-based regimens are now recommended first-line regimens by all major treatment guidelines and are available as single-tablet regimens (EVG/COBI/FTC/TDF; Stribild^®^, EVG/COBI/FTC/TAF; Genvoya^®^, and DTG/ABC/3TC; Triumeq^®^) [31, 33–35]. However, as with other antiretrovirals, resistance to INSTIs develops through the selection of specific mutations in the integrase gene. Although some minor/secondary INSTI RAMs may pre-exist, major/primary INSTI RAMs have been rarely detected in treatment-naive patients with few reports of INSTI transmitted drug resistance [15, 36–39]. Therefore, genotypic testing for pre-existing drug resistance mutations in the integrase gene is currently recommended only if transmitted drug resistance against INSTIs is a concern [16, 17].

**Fig 1:**
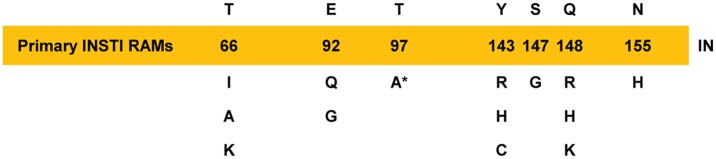
Emergent primary INSTI resistance-associated mutations (RAMs). Commonly observed major/primary INSTI RAMs against EVG and/or RAL are shown at indicated integrase positions below the yellow bar. Resistance-associated mutations were based on IAS-USA Guidelines [17] with some modifications (see Methods). *, T97A may require additional integrase mutations for reduced phenotypic susceptibility to EVG and/or RAL.

The genetic barrier to antiretroviral drug resistance depends on the ease with which genetic change(s) occur and their selective phenotypic advantage(s) during antiretroviral therapy [40]. During virologic failure, some mutations persist while others emerge as HIV-1 evolves towards greater resistance and improved viral fitness [41, 42]. As such, the emergence of INSTI resistance is defined by one or more non-polymorphic primary INSTI RAMs that compromise INSTI susceptibility (Fig 1) [43–45] and possible secondary INSTI RAMs that may have no effect or may further reduce INSTI susceptibility and/or improve viral replication capacity [44, 46–50]. In general, most secondary INSTI RAMs are absent or extremely rare in INSTI-naive patients. Other secondary INSTI RAMs occur as natural integrase polymorphisms with different prevalence according to different HIV-1 subtypes and prior antiretroviral drug exposure [51–63]. Although individual integrase polymorphisms generally have little or no effect on INSTI susceptibility [50, 64, 65], there is a concern that subtype-specific resistance-associated integrase polymorphisms could facilitate viral evolution of resistance under INSTI pressure [44, 52, 56–58, 60, 61, 66–71].

The T97A integrase mutation is an HIV-1 integrase polymorphism of interest to clinicians considering INSTI-based therapy for HIV-infected patients under their care. Depending on subtype, T97A generally pre-exists in 1–5% of INSTI-naive HIV-infected individuals with some exceptions (subtype A: 5–10%; subtype J: 33%; group P: 50%) [53, 72], yet has also been associated with emergent INSTI resistance in patients experiencing virologic failure on RAL or EVG. However, despite the fact that some patients may harbor T97A before INSTI-based treatment, little is known about the clinical and prognostic implications of this naturally occurring resistance-associated integrase polymorphism.

Across multiple clinical studies, T97A has been periodically co-selected in the presence of primary INSTI RAMs by RAL [25, 32, 41, 43, 66, 73–78] and EVG [25, 79]. T97A has also been selected by DTG in TE patients with pre-existing primary RAL RAMs [80, 81]. As a secondary INSTI RAM, T97A further reduces INSTI susceptibility and/or rescues viral fitness in association with Y143C/R [41, 47, 82], Q148H+G140S [83], or N155H [67, 73, 84]. In rare cases, T97A has also been selected alone (i.e., in the absence of primary INSTI RAMs) by RAL [85–88] and EVG [25, 89], which led us to previously consider T97A alone as a special case of primary INSTI RAM that requires additional integrase mutations (Fig 1) [25, 45, 89]. Indeed, while T97A alone confers low-level to no effect on EVG and RAL susceptibility, respectively [45, 50], additional integrase polymorphism(s) such as V72I, L74M, and/or G163R may serve a role to further reduce INSTI susceptibility before or during INSTI-based treatment [86–88]. Whether the appearance of T97A is natural or transmitted, it is currently unclear if HIV-infected patients with pre-existing T97A are at risk to delayed virologic suppression or earlier virologic failure and the development of INSTI resistance. This is particularly important as T97A could change the guideline recommendation for pre-treatment integrase genotyping to include all patients, regardless of suspected INSTI transmitted drug resistance.

In this study, the prevalence of T97A was evaluated as a pre-existing or emergent integrase mutation relative to its impact on INSTI susceptibility and treatment outcome in HIV-infected patients enrolled on EVG- or RAL-based regimens.

## Methods

### Ethics statement

Written informed consent was obtained from all patients prior to any study-related procedures. Clinical investigation in each of the following previously published clinical studies was conducted according to the principles expressed in the Declaration of Helsinki and the guidelines of the International Conference on Harmonisation Good Clinical Practice. As indicated below, these studies were conducted at multiple study centers/sites in multiple countries and were accordingly approved by a central institutional review board (Chesapeake IRB, Columbia, MD) and site-specific institutional review boards/ethics committees.

Patient data was selected from 16 phase 1, 2 or 3 clinical studies previously reviewed and approved by the US Department of Health and Human Services or the European Medicines Agency with the following clinical trial (CT) identifiers: GS-US-01-934 (NCT00112047; 59 sites; 6 countries) [90], GS-US-99-903 (NCT00158821; 13 sites; 3 countries) [91], GS-US-99-907 (NCT00002450; 72 sites; 8 countries) [92], GS-US-183-0105 (NCT00298350; 78 sites; 1 country) [24] [93], GS-US-183-0130 (NCT00445146; 59 sites; 1 country), GS-US-183-0145 (NCT00708162; 234 sites; 13 countries) [25], GS-US-183-0152 (EudraCT2008-003917-29; 10 sites; 3 countries) [94], GS-US-236-0102 (NCT01095796; 102 sites; 1 country) [95, 96], GS-US-236-0103 (NCT01106586; 148 sites; 16 countries) [27, 89], GS-US-236-0104 (NCT00869557; 30 sites; 1 country) [97], GS-US-236-0118 (NCT01363011; 40 sites; 8 countries) [98], GS-US-236-0128 (NCT01705574; 80 sites; 11 countries) [99], GS-US-292-0102 (NCT01497899; 37 sites; 1 country) [100, 101], GS-US-292-0104 (NCT01780506; 120 sites; 11 countries) [26, 101], GS-US-292-0106 (NCT01854775; 10 sites; 4 countries) [102, 103], GS-US-292-0111 (NCT01797445; 121 sites; 10 countries) [26, 101].

### Study population and design

Eligible patients were required to be INSTI-naive with screening plasma HIV-1 RNA of at least 500 copies/mL (COBAS Amplicor HIV-1 Monitor Test, v1.5; Roche Diagnostics, Basel, Switzerland). Upon enrollment, clinical data (i.e., HIV-1 RNA, CD4 cell count, adherence by pill count in returned pill bottles) were evaluated was monitored on-treatment every 4–12 weeks up to or beyond protocol-defined primary or secondary efficacy endpoints (Weeks 48, 96, or 144). INSTI-naive and INSTI-treated patients received, in their optimized regimen, at least one NRTI, one boosted PI, and/or one NNRTI plus, for some of them, maraviroc (MVC) or enfuvirtide (ENF).

Sequences of the entire integrase gene derived from HIV-infected patients screened and/or enrolled across multiple clinical studies (Table 1) were analyzed for pre-existence or emergence of the T97A mutation using the HIV-1_NL4-3_ (subtype B) integrase sequence as a reference. Pre-treatment data selected from INSTI-naive patients was inclusive of all clinical studies, irrespective of enrollment on an INSTI-based regimen. On-treatment data selected from INSTI-treated patients was inclusive of clinical study cohorts in which patients enrolled on an INSTI-based regimen that contained clinically approved or bioequivalent doses of EVG (85/125/150 mg once a day) or RAL (400 mg twice a day). INSTI-treated patients were either highly antiretroviral treatment-experienced (TE) with 1–2 class resistance and enrolled on ritonavir-boosted (/r) EVG once a day or RAL twice a day plus an active PI and an active or inactive second antiretroviral (1 NRTI; 1 or 2 among etravirine (ETR), MVC, or ENF) [Studies 183-0105/0130/0145/0152] or INSTI-treated patients were antiretroviral treatment-naive (TN) and enrolled on cobicistat-boosted (/COBI) EVG once a day as part of a fixed-dose combination with FTC/TDF (Stribild^®^) [Studies 236-0102/0103/0104/0118/0128 and 292-0104/0111] or FTC/TAF (Genvoya^®^) [Studies 292-0102/0104/0106/0111].

**Table 1:** Cross-study frequency of pre- and on-treatment integrase genotypes with or without T97A mutation.

Study Drug(s)^a^	Study No.	PT POP^b^	Pre-Treatment			On-Treatment^c^	
			INSTI Treatment Arm	Integrase Genotype	T97A; Pre-Existing (Enrolled on INSTI)	Primary INSTI RAMs	T97A Alone^d^ (Emergent)
**TDF**	01-934/ 99–903	TN	0	200	1 (0)	-	-
	99–907	TE	0	110	0	-	-
**EVG (125 mg)/r+OBR**	183–0105	TE	73	277	0	37	0
**EVG (85/150 mg)/r+BR**	183-0130/0145/ 0152	TE	375	164	3 (3)	30	6 (5)
**RAL (400 mg)+BR**	183–0145	TE	351	152	0	26	2 (2)
**EVG (150 mg)/ COBI/FTC/TDF**	236-0102/0103/ 0104/0118/ 0128	TN	1071	475	17 (14)	15	1 (1)
**EVG (150 mg)/ COBI/FTC/TAF or EVG (150 mg)/ COBI/FTC/TDF**	292-0102/0104/ 0106/ 0111	TN	2011	1989	26 (1)	14	0
**Total:**			**3881**	**3367**	**47 (18)**	**122**	**9 (8)**

BR, background regimen; OBR, optimized background regimen; PT POP, patient population; TE, treatment-experienced; TN, treatment-naive; INSTI, integrase strand transfer inhibitor; TDF, tenofovir disoproxil fumarate; EVG, elvitegravir; RAL, raltegravir

^a^ OBR included ≥2 NRTIs ± ENF with documented ≥1 PI mutation(s). BR included active PI + active/inactive 2nd agent (NRTI, ETR, MVC, ENF) with EVG 85 mg once a day (ATV/r or LPV/r), EVG 125 mg once a day (DRV/r, FPV/r, or TPV/r), or RAL 400 mg twice a day.

^b^ Patients were either highly antiretroviral treatment-experienced (INSTI-naive) with 1–2 class resistance or antiretroviral treatment-naive (INSTI-naive).

^c^ Only patients that received clinically approved EVG 150 mg once a day, bioequivalent EVG 85/125 mg once a day, or RAL 400 mg twice a day were included in on-treatment analysis of integrase genotypes.

^d^ Alone, defined as in the absence of primary INSTI RAM(s)

### Genotypic and phenotypic resistance analysis

Integrase genotyping and phenotyping of plasma samples was performed at Monogram Biosciences (South San Francisco, CA) using the GeneSeq^™^ Integrase or GenoSure^™^ Integrase (IN) and Phenosense^™^ Integrase assays, respectively. Primary INSTI RAMs against EVG and/or RAL were based on IAS-USA Guidelines of RAMs [17] with some modifications to remove secondary INSTI RAMs (i.e., L74M, F121Y, E138A/K, and G140A/S) (Fig 1). Reductions in INSTI susceptibility were represented as fold changes (FC) in EC_50_ value (effective concentration that results in 50% inhibition) of the patient-derived test virus relative to the EC_50_ value of HIV-1_NL4-3_ (subtype B) reference virus. Biological cutoffs were defined as the 99^th^ percentile of the distribution of FC EC_50_ values of INSTI-naive patients above which reduced INSTI susceptibility may be interpreted. The biological cutoffs used by Monogram Biosciences in the Phenosense^™^ Integase assay are as follows: EVG, ≥2.5; RAL, ≥1.5; and DTG, ≥2.5 [88, 104].

Resistance testing of pre-treatment plasma samples occurred prospectively or retrospectively for INSTI-naive patients at screening or baseline visits, according to individual study design or ad hoc analyses. Pre-treatment analyses of integrase sequences were inclusive of all available patient data with on-treatment analyses restricted to patients enrolled on EVG 85/125/150 mg once a day or RAL 400 mg twice a day.

Resistance testing of on-treatment plasma samples occurred prospectively for INSTI-treated patients with HIV-1 RNA ≥400 copies/mL at the confirmation of virologic failure visit or last visit. Protocol-defined virologic failure was either suboptimal virologic response (HIV-1 RNA <1 log_10_ decrease from baseline and ≥50 copies/mL by Week 8 and confirmed at the Week 12 visit) or virologic rebound (HIV-1 RNA ≥400 copies/mL after achieving <50 copies/mL or having 2 consecutive visits with >1 log_10_ increase in HIV-1 RNA from nadir). Virologic rebound required a confirmation unless the rebound occurred at protocol-defined primary or secondary efficacy endpoints (Week 48, 96, or 144), early study drug discontinuation, or last visit.

On-treatment analyses of integrase sequences considered only the first confirmed protocol-defined virologic failure visit with persistent (no change from pre-treatment integrase sequence) or emergent (change from pre-treatment integrase sequence) T97A integrase mutation, irrespective of preceding or concurrent resistance to other co-administered antiretrovirals. All patients with the T97A integrase mutation at protocol-defined virologic failure visit(s) have been previously reported in study-specific resistance analysis populations [25, 89]. All patients that withdrew consent and/or had the T97A integrase mutation at a non-protocol-defined virologic failure visit(s) have been excluded from previously reported efficacy analyses and thus were excluded from treatment outcome analyses described in this report. However, due to the limited number of patients with the T97A integrase mutation, all patients were included in phenotypic resistance analyses irrespective of protocol-defined status. Finally, previously reported resistance to other antiretroviral classes has been excluded from the main body of this report and is discussed anecdotally (S1–S3 Tables and S1–S3 Figs).

### Statistical analyses

Fisher’s exact test was used to compare the frequency of the T97A integrase mutation between patient populations. Student’s *t*-test was used to compare mean FC EC_50_ in EVG susceptibility between patient populations. All differences with a *P* values of <0.05 were considered statistically significant.

## Results

### Prevalence of pre-existing T97A was infrequent among INSTI-naive (treatment-experienced and treatment-naive) patients

**Fig 2:**
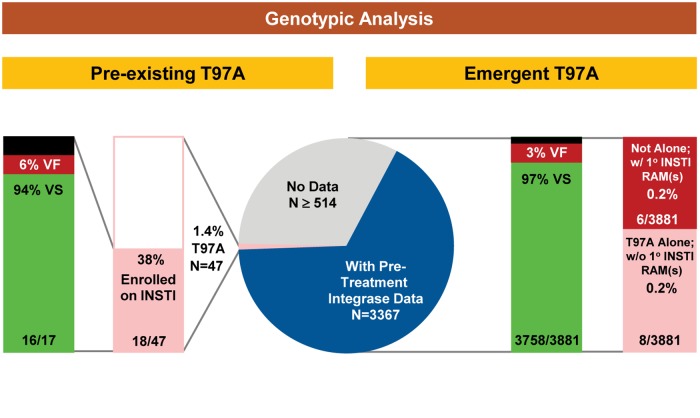
Pre- and on-treatment incidence of HIV-1 infected patients with T97A-based genotypic EVG resistance and impact on treatment outcome. Grey indicates no data; black indicates excluded data; green indicates virologic success (VS), red indicates virologic failure (VF). **Left panel**: Forty-seven patients had pre-existing T97A, of which 18 patients (TE = 3; TN = 15) enrolled on an INSTI-based regimen: 16 patients (1 TE [on EVG/r+TDF+DRV] and 15 TN [on EVG/COBI/FTC/TDF]) achieved HIV-1 RNA <50 copies/mL by study endpoint and were considered VS, 1 patient (1 TE [on EVG/r+TDF+DRV]) did not achieve HIV-1 RNA <50 copies/mL by a non-protocol-defined virologic failure visit (Week 8) and was excluded from treatment outcome and study-specific resistance analysis (see Methods), and 1 patient (1 TE [on EVG/r+TDF+DRV]) maintained T97A and EVG sensitivity at confirmed virologic rebound (Week 40) without further development of antiretroviral resistance. **Right panel:** Eight patients (TE = 7; TN = 1) had emergent T97A alone (ie, in the absence of primary INSTI RAMs): 4 patients at first protocol-defined virologic failure (3 TE [1 on RAL+ABC+LPV/r, 1 on EVG/r+FTC/TDF+LPV, and 1 on EVG/r+MVC+DRV]; 1 TN [on EVG/COBI/FTC/TDF]), 2 patients at second protocol-defined virologic failure (2 TE [1 on EVG/r+TDF+DRV and 1 on RAL+ABC+LPV/r]), 1 patient at fourth protocol-defined virologic failure (1 TE [on EVG/r+TDF+DRV]), and 1 patient (1 TE [on EVG/r+ETR+LPV]) withdrew consent and discontinued study drug at Week 144 and was excluded from treatment outcome and study-specific resistance analysis (see Methods).

Altogether, 3881 INSTI-naive patients were enrolled in clinical studies on an INSTI-based regimen, and received at least 1 dose of study drugs (Table 1 and Fig 2). Across all studies, most patients received a clinically approved or bioequivalent dose of EVG 85/125/150 mg once a day (3530 of 3881; 91%), while the remaining patients (all from Study 183–0145) received RAL 400 mg twice a day (351 of 3881; 9%). The majority of patients (3082 of 3881; 79%) were TN and enrolled on a fixed-dose combination of EVG with COBI, FTC, and TDF or TAF (EVG/COBI/FTC/TDF or EVG/COBI/FTC/TAF). The remaining patients (799 of 3881; 21%) were highly TE, but INSTI treatment-naive, with pre-existing antiretroviral resistance and enrolled on ritonavir-boosted EVG or RAL, each administered with a background regimen containing a fully active ritonavir-boosted PI and an active or inactive second agent. As part of study-specific prospective screening requirements or retrospective ad hoc baseline analyses, INSTI-naive patients were analyzed for pre-existing resistance in integrase, including patients that did not enroll on INSTI-based treatment arms.

Of the 3881 INSTI-treated patients, a subpopulation of 3367 individual pre-treatment integrase sequences were obtained and analyzed (2664 TN patients and 703 TE patients). None of these pre-treatment integrase sequences contained primary integrase mutations most often associated with emergent EVG (T66I, E92Q, S147G, Q148R/H/K, and N155H) or RAL (Y143C/R/H, Q148H/K/R, and N155H) resistance. Some secondary integrase mutations associated with resistance to EVG and/or RAL were detected as natural integrase polymorphisms, most often at very low frequencies (0.5–1%: V72T, L74M, A128T, and G163R) with few exceptions (19%: M50I; 59%: S119P/G/T/R; and 3.8%: E157Q).

The T97A (or T97T/A) integrase mutation was detected as an infrequent, naturally occurring integrase polymorphism among all available pre-treatment integrase sequences in 1.4% of patients (47 of 3367) (Table 1 and Fig 2; S1 Table and S1 Fig). Of the total number of pre-existing T97A (or T97T/A) integrase mutations detected, a slightly greater proportion were detected as full mutants (26 of 47 patients; 55%) than wild-type mixtures (21 of 47; 45%). Consistent with the overall distribution of patients, pre-treatment integrase sequences harboring T97A were unevenly divided among TN (44 of 47 patients; 94%) and TE patients (3 of 47 patients; 6%). As a proportion of the total pre-treatment integrase genotypes analyzed, T97A was less frequently detected in TE patients (3 of 703 patients; 0.4%) than TN patients (44 of 2664 patients; 1.7%)(*P* = 0.01). Finally, 38% (18 of 47) of patients with pre-existing T97A enrolled onto an INSTI-based regimen (15 TN and 3 TE).

### Pre-existing T97A was more prevalent among non-B subtypes

Of the total pre-treatment IN genotypes analyzed, most patients had HIV-1 subtype B (2826 of 3238; 87%) which was consistent with enrollment demographics across the majority of the studies in North America and Europe (Table 2). The remaining proportion of non-B subtypes (412 of 3238; 13%) provided in this data set included subtypes A (0.3%), A1 (1.6%), A2 (0.1%), AE (4.8%), AG (1.6%), C (1.4%), D (0.6%), F1 (0.3%), G (0.5%), and complex (1.4%).

**Table 2:** HIV-1 subtype frequency among pre- and on-treatment integrase genotypes with or without T97A integrase mutation.

HIV-1 Subtype, N (%)	Pre-Treatment (% Integrase genotypes; % Total subtype)			On-Treatment (% Integrase genotypes)	
	Total	Pre-Existing T97A	Pre-Existing T97A; Enrolled on INSTI	Primary INSTI RAMs	Emergent T97A Alone^a^
**Integrase genotypes (with subtype data)**	3238	47	18	121	8
**B**	2826 (87%)	25 (53%; 0.9%)	8 (44%; 0.3%)	117 (97%)	8 (100%)
**Non-B**	412 (13%)	22 (47%; 5.3%)	10 (56%; 2.4%)	4 (3%)	0
**A**	9 (0.3%)	3 (6.4%; 33%)	2 (11%; 22.2%)	0	0
**A1**	53 (1.6%)	9 (19%; 17%)	4 (22%; 7.5%)	1 (1%)	0
**A2**	2 (0.1%)	0	0	0	0
**AE**	156 (4.8%)	1 (2.1%; 0.6%)	0	0	0
**AG**	51 (1.6%)	2 (4.3%; 3.9%)	0	2 (2%)	0
**C**	45 (1.4%)	0	0	0	0
**D**	21 (0.6%)	4 (8.5%; 19%)	2 (11%; 9.5%)	0	0
**F1**	11 (0.3%)	0	0	1 (1%)	0
**G**	17 (0.5%)	2 (4.3%; 12%)	2 (11%; 11.8%)	0	0
**Cpx**	46 (1.4%)	1 (2.1%; 2.2%)	0	0	0

Cpx, complex

^a^ Alone, defined as in the absence of primary INSTI RAM(s)

While T97A was infrequently detected among pre-treatment integrase genotypes, T97A was more prevalent in patients harboring non-B (22 of 412; 5.3%) than B (25 of 2826; 0.9%) subtypes (*P* < 0.001). Among non-B subtypes, the frequency of T97A was highest among subtype A (33%), A1 (17%), D (19%), and G (11.8%) variants, lowest among subtype AE (0.6%), AG (3.9%), and complex (2.2%) variants, and not detected among subtype A2, C, and F1 variants. Of the 47 patients with pre-existing T97A, the proportion of B (25 of 47; 53%) and non-B (22 of 47; 47%) subtypes were roughly equivalent. Finally, more non-B (10 of 18; 56%) than B (8 of 18; 44%) patients harboring pre-existing T97A were enrolled onto an INSTI-based regimen.

### Codon usage at integrase amino acid position 97 among B and non-B subtypes

Codon usage at integrase amino acid position 97 was examined from a 4-fold larger data set of patient-derived integrase sequences to define its relation to the prevalence of T97A among non-B subtypes. A total of 13455 integrase sequences harboring the same viral subtypes identified in the above clinical studies were queried from the Los Alamos HIV database (http://www.hiv.lanl.gov/components/sequence/HIV/search/search.html) (Table 3). Although some of these integrase sequences may have originated from patients on an INSTI-based regimen, the vast majority (65% on average) pre-dated the approval of the first INSTI (RAL) in 2007.

**Table 3:** Analysis of codon usage at integrase amino acid position 97 among patient-derived integrase sequences queried from the Los Alamos HIV database.

HIV-1 Subtype, N	Total	Codon Usage at Integrase Amino Acid Position 97^a^								
		T97				A97	S97	P97	I97	V97
		ACA	ACG	ACC	ACT	GCA	TCA	CCA	ATA	GTA
**Integrase genotypes (with subtype data)**	13455	13016 (97%)	81 (0.6%)	4 (<0.1%)	43 (0.3%)	255 (1.9%)	47 (0.3%)	2 (<0.1%)	2 (<0.1%)	4 (<0.1%)
**B**	5330	5258 (99%)	14 (0.3%)	0	19 (0.4%)	37 (0.7%)	1 (<0.1%)	1 (<0.1%)	0	0
**Non-B**	8125	7758 (96%)	67 (0.8%)	4 (<0.1%)	24 (0.3%)	218 (2.7%)	46 (0.6%)	1 (<0.1%)	2 (<0.1%)	4 (<0.1%)
**A**	54	47 (86%)	0	0	0	5 (9.3%)	1 (1.9%)	0	0	0
**A1**	1271	1089 (86%)	4 (0.3%)	0	2 (0.2%)	159 (12.5%)	12 (0.9%)	0	1 (0.1%)	4 (0.3%)
**A2**	26	26 (100%)	0	0	0	0	0	0	0	0
**AE**	2252	2232 (99%)	15 (0.7%)	1 (<0.1%)	1 (<0.1%)	3 (0.1%)	0	0	0	0
**AG**	686	641 (93%)	4 (0.6%)	2 (0.3%)	2 (0.3%)	37 (5.4%)	0	0	0	0
**C**	2544	2482 (98%)	20 (0.8%)	0	9 (0.4%)	1 (<0.1%)	31 (1.2%)	0	1 (<0.1%)	0
**D**	765	737 (96%)	21 (2.7%)	0	5 (0.7%)	0	1 (0.1%)	1 (0.1%)	0	0
**F1**	107	98 (92%)	2 (1.9%)	1 (0.9%)	4 (3.7%)	2 (1.9%)	0	0	0	0
**G**	270	259 (96%)	0	0	1 (0.4%)	9 (3.3%)	1 (0.4%)	0	0	0
**Cpx**	150	147 (98%)	1 (0.7%)	0	0	2 (1.3%)	0	0	0	0

cpx, complex

^a^ Only codons observed are shown

Overall, 99% (5258 of 5330) of subtype B and 96% (7758 of 8125) of non-B variants harbored ACA at position 97, consistent with a high prevalence of T97. Among non-B variants, the usage of codon ACA at position 97 was lowest in both subtype A (47 of 54; 86%) and A1 (1089 of 1271; 86%) variants. The 3 other threonine codons (ACG, ACC, and ACT) were observed at <1% frequency. All remaining integrase sequences harbored low-frequency integrase polymorphisms at amino acid position 97 (described below). Each polymorphism was represented by only a single codon, consistent with single nucleotide changes of the predominant wild-type codon for threonine.

T97A was the most prevalent low frequency integrase polymorphism detected as GCA at position 97 (255 of 13455; 1.9%), consistent with an ACA-to-GCA transition. Similar to our pre-treatment analysis, T97A was more frequently detected in the Los Alamos HIV database among HIV-1 non-B (218 of 8125; 2.7%) than B (37 of 5330; 0.7%) subtypes (*P* < 0.001). Among non-B variants, the frequency of T97A was highest in both subtype A (5 of 54; 9.3%) and A1 (159 of 1271; 12.5%) variants. The frequency of T97A among non-B variants in this larger data set was generally lower than in our pre-treatment data set (Table 2).

Other polymorphisms at position 97 were also detected at very low frequencies. T97S was detected as TCA at position 97 (47 of 13455; 0.3%), consistent with an ACA-to-TCA transversion. The frequency of T97S (31 of 2544; 1.2%) was greater than T97A (1 of 2544; <0.1%) in subtype C variants. Other polymorphisms detected at frequencies <0.1% included T97P (ACA-to-CCA transversion), T97I (ACA-to-ATA transition), and A97V (GCA-to-GTA transition).

### Emergence of T97A was infrequent among INSTI-treated (treatment-experienced and treatment-naive) patients

Overall, 97% (3758 of 3881) of INSTI-treated patients (EVG: 3433 of 3530, 97%; RAL: 325 of 351, 93%) achieved virologic success and maintained HIV-1 RNA <50 copies/mL through protocol-defined primary or secondary efficacy endpoints (Week 48, 96, or 144) (Table 1 and Fig 2). Correspondingly, resistance development to EVG or RAL occurred infrequently in 3% (122 of 3881) of INSTI-treated patients (EVG: 96 of 3530, 2.7%; RAL: 26 of 351, 7.4%). Among on-treatment integrase sequences harboring emergent primary INSTI RAMs, a greater proportion were derived from TE patients enrolled on a regimen containing ritonavir-boosted EVG or RAL (92 of 122; 75%) than TN patients enrolled on a fixed-dose combination containing cobicistat-boosted EVG (30 of 122; 25%). Patients with emergent EVG or RAL resistance often harbored a single primary INSTI RAM, although a complex dynamic mixture of up to 4 mutations were occasionally observed (data not shown) [25, 89, 93, 96, 101, 103].

Among the few patients that experienced virologic failure with INSTI RAMs, the T97A integrase mutation was found to emerge infrequently (11%; 14 of 122). In 6 of these 14 patients (EVG, n = 4; RAL, n = 2), the T97A (or T97T/A) integrase mutation emerged in the presence of primary INSTI RAM(s) (N155H, n = 5; E92Q, n = 1) and all had HIV-1 subtype B (Table 1 and Fig 2; S2 Table and S2 Fig). In the remaining 8 patients (EVG, n = 6; RAL, n = 2), the T97A (or T97T/A) integrase mutation emerged alone, in the absence of primary INSTI RAMs (Table 1 and Fig 2; S3 Table and S3 Fig). Among these 8 patients with emergent T97A alone, 4 patients co-developed a secondary INSTI RAM: V72N (1 TE patient on EVG/r+TDF+DRV), L74M (2 TE patients on RAL+ABC+LPV/r), and G163R (1 TN patient on EVG/COBI/FTC/TDF). In addition, 7 patients harbored ≥1 secondary INSTI RAM(s) that persisted from baseline: M50I (n = 2), S119G (n = 1), S119P (n = 3), and S119R (n = 2).

All 8 patients (1 TN and 7 TE) with emergent T97A alone (i.e., in the absence of primary INSTI RAMs) had HIV-1 subtype B. Of these, 50% (4 of 8) of patients developed T97A in year 1 and 50% (4 of 8) of patients developed T97A at a second or subsequent protocol-defined virologic failure visit in year 2 or 3. The 1 TN patient with emergent T97A in year 3 (from Study 236–0103) co-developed NRTI (M184V) resistance on EVG/COBI/FTC/TDF at their first protocol-defined virologic failure visit and then later regained virologic suppression by Week 144 at which point study drug was discontinued [89]. Of the 7 TE patients with emergent T97A alone (all from Study 183–0145), none developed additional resistance in protease or reverse transcriptase [25].

To assess potential predictors of T97A emergence in the absence of primary INSTI RAMs, pre-treatment characteristics of HIV-1 RNA and CD4 cell count and on-treatment characteristics of adherence by pill count in returned pill bottles were evaluated (S3 Table). Among the 8 patients with emergent T97A alone (1 TN and 7 TE), 13% (1 of 8; 1 TN) of patients had baseline HIV-1 RNA >100,000 copies/mL, 38% (3 of 8; 2 TE and 1 TN) of patients had baseline CD4 count <200 cells/mL, and 63% (5 of 8; 4 TE and 1 TN) of patients had adherence rates <95%. All 7 TE patients with emergent T97A alone harbored pre-existing resistance mutations to 1 (n = 3), 2 (n = 2), or 3 (n = 2) antiretroviral classes. However, only 28% (2 of 7) of these patients harbored resistance mutations that were predicted to reduce phenotypic susceptibility to antiretrovirals in their background regimen (HIVdb algorithm; Stanford, CA) [105].

### Patient-derived viruses containing T97A alone may exhibit low-level reductions in susceptibility to EVG and/or RAL

**Fig 3:**
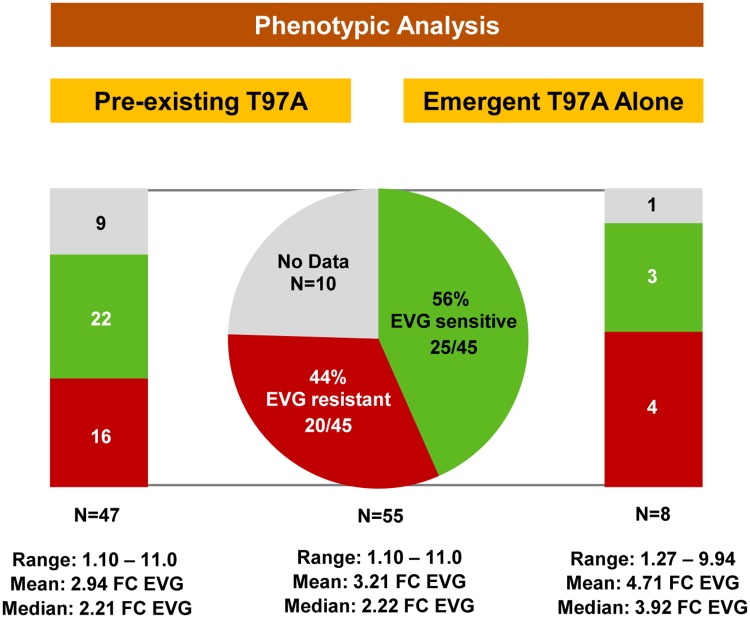
Pre- and on-treatment incidence of HIV-1 infected patients with T97A-based phenotypic EVG resistance. Grey indicates no data; black indicates excluded data, green indicates EVG susceptibility (i.e., EVG sensitive), red indicates reduced EVG susceptibility (i.e., EVG resistant). **Left panel:** Forty-seven patients had pre-existing T97A: 16 patients (9 enrolled on INSTI: 1 TE [on EVG+TDF+DRV/r] and 8 TN [on EVG/COBI/FTC/TDF]) with reduced EVG susceptibility, 22 patients (9 enrolled on INSTI: 2 TE [on EVG/r+TDF+DRV] and 7 TN [on EVG/COBI/FTC/TDF]) with EVG sensitivity, and 9 patients with no phenotypic data. **Right panel:** Eight patients (TE = 7; TN = 1) had emergent T97A alone (i.e., in the absence of primary INSTI RAMs): 4 patients (4 TE [2 on RAL+ABC+LPV/r, 1 on EVG/r+MVC+DRV, and 1 on EVG/r+TDF+DRV]) with reduced EVG susceptibility, 3 patients (3 TE [1 on EVG/r+TDF+DRV, 1 on EVG/r+FTC/TDF+LPV, and 1 on EVG/r+ETR+LPV]) with EVG sensitivity including 1 patient (1 TE [on EVG/r+ETR+LPV]) that withdrew consent and discontinued study drug at Week 144 and was excluded from treatment outcome and study-specific resistance analysis (see Methods), and 1 patient (1 TN [on EVG/COBI/FTC/TDF]) with no phenotypic data. The proportion of patients with reduced EVG susceptibility was low and similar between pre- and on-treatment T97A populations (*P* = 0.682).

Altogether, 55 patient-derived viruses harboring T97A (or T97T/A) alone (i.e., in the absence of primary INSTI RAMs) were tested for phenotypic susceptibility to INSTIs, with data available for 45 isolates (pre-existing T97A: 38 of 47; emergent T97A: 7 of 8) (Table 1 and Fig 3; S1 and S3 Tables, S1 and S3 Figs). Reduced susceptibility to EVG (20 of 45; 44%) and/or RAL (17 of 45; 38%) was generally uncommon, while susceptibility to DTG was maintained against all samples tested (data not shown). Overall, fold change EC_50_ values to EVG and RAL were low and varied broadly from 1.10 to 11.0 for EVG (mean FC = 3.21; median FC = 2.22) and from 0.60 to 4.04 for RAL (mean FC = 1.50; median FC = 1.30).

**Fig 4:**
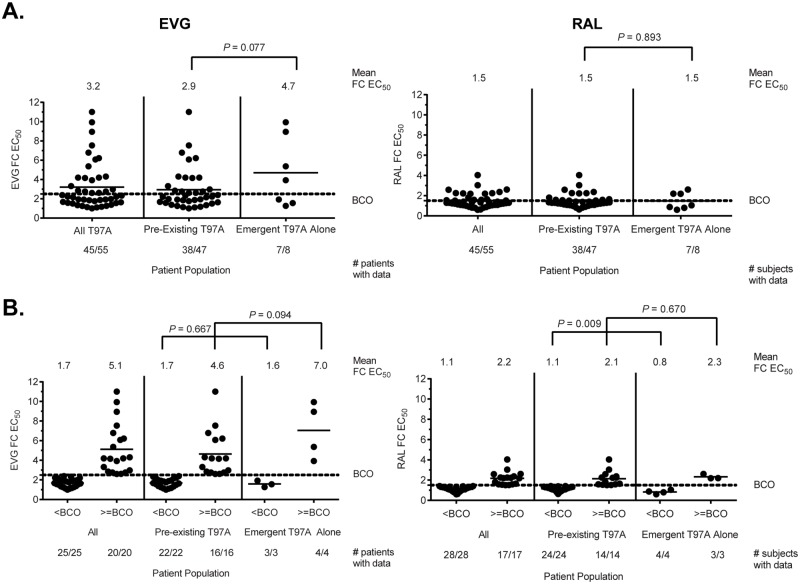
Overall fold change differences in EVG and RAL susceptibility between pre-existing and emergent T97A alone populations. Fifty-five patient-derived viruses harboring T97A (or T97T/A) alone (i.e., in the absence of primary INSTI RAMs) were tested for phenotypic susceptibility to INSTIs, with data available for 45 isolates (pre-existing T97A: 38 of 47; emergent T97A alone: 7 of 8). All isolates maintained sensitivity to DTG (data not shown). Fold change EC_50_ values against EVG (**left panel**) or RAL (**right panel**) were compared between overall pre-existing and emergent T97A alone populations **(A)** and between pre-existing and emergent T97A alone populations with reduced susceptibility greater than or equal to biological cutoff (BCO) thresholds **(B)**. Biological cutoff fold-change (FC) EC_50_ values for EVG and RAL are ≥2.5 and ≥1.5, respectively (PhenoSense^™^ Integrase assay). Statistical significance was calculated using a two-tailed Student’s t-test assuming equal variance.

No significant differences in EVG or RAL susceptibility were observed between overall pre-existing and emergent T97A populations (*P* = 0.077 and *P* = 0.893, respectively) or between pre-existing and emergent T97A populations with reduced EVG or RAL susceptibility greater than biological cutoffs (*P* = 0.094 and *P* = 0.670, respectively)(Fig 4A and 4B). Finally, there was no difference between the proportion of patients with reduced EVG susceptibility and the proportion of patients with reduced RAL susceptibility in both the pre-existing and emergent T97A populations (*P* = 0.682 and *P* = 1.00, respectively).

**Fig 5:**
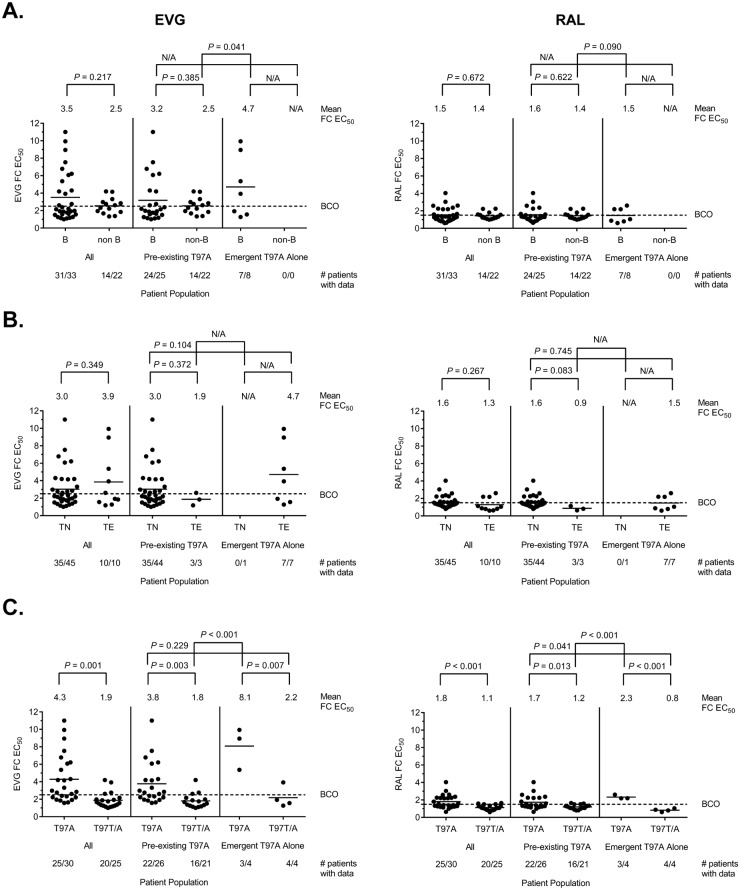
Stratified fold change differences in EVG and RAL susceptibility between and among pre-existing and emergent T97A alone populations. Fifty-five patient-derived viruses harboring T97A (or T97T/A) alone (i.e., in the absence of primary INSTI RAMs) were tested for phenotypic susceptibility to INSTIs, with data available for 45 isolates (pre-existing T97A: 38 of 47; emergent T97A alone: 7 of 8). All isolates maintained sensitivity to DTG (data not shown). Fold change EC_50_ values against EVG (**left panel**) or RAL (**right panel**) were compared between and among overall pre-existing and emergent T97A alone populations by B versus non-B subtype **(A)**, by TN versus TE **(B)**, and by full T97A versus wild-type mixed T97T/A mutation **(C)**. Biological cutoff (BCO) fold-change (FC) EC_50_ values for EVG and RAL are ≥2.5 and ≥1.5, respectively (PhenoSense^™^ Integrase assay). Statistical significance was calculated using a two-tailed Student’s t-test assuming equal variance.

Overall, no fold change differences in EVG or RAL susceptibility were observed between B and non-B subtype variants (*P* = 0.217 and *P* = 0.672, respectively) or between TN and TE patients (*P* = 0.349 and *P* = 0.267, respectively), with similar findings within or between the pre- and on-treatment T97A patient populations (Fig 5A and 5B). However, fold change differences in EVG or RAL susceptibility were observed between isolates harboring T97A and T97T/A variants (*P* = 0.001 and *P* < 0.001, respectively), with similar findings within the pre- and on-treatment T97A patient populations (Fig 5C). Taken together, these results indicate that pre-existence or emergence of T97A alone with reduced susceptibility to EVG and/or RAL is infrequent and not strongly associated with drug selection pressure, subtype, or prior antiretroviral exposure.

**Fig 6:**
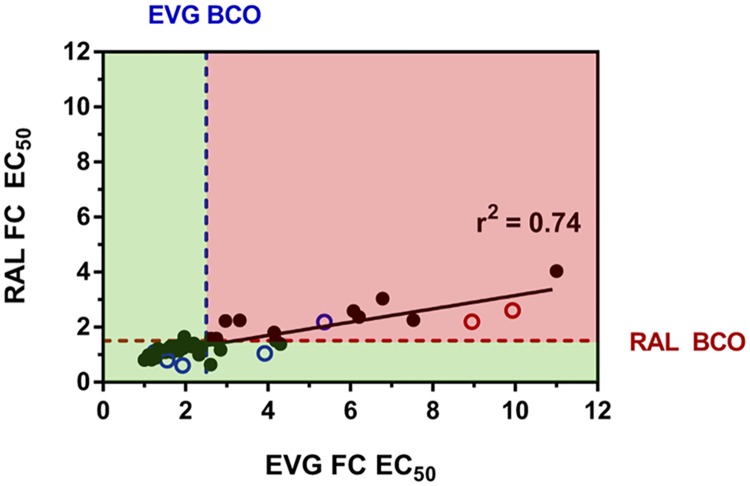
Correlation of EVG and RAL susceptibility among patient-derived viruses containing T97A alone (pre-existing and emergent). Solid black circles represent pre-existing T97A isolates at pre-treatment visits (38 of 47 with data). Open circles represent emergent T97A alone isolates (i.e., in the absence of primary INSTI RAMs) at first protocol-defined virologic failure visits with INSTI resistance (7 of 8 with data); blue and red indicate EVG and RAL-based regimens, respectively. Biological cutoff fold-change (FC) EC_50_ values for EVG and RAL are ≥2.5 and ≥1.5, respectively (PhenoSense^™^ Integrase assay). Fold-change EC_50_ values were low and varied broadly from 1.10 to 11.0 for EVG (mean FC = 3.21; median FC = 2.22) and from 0.60 to 4.04 for RAL (mean FC = 1.50; median FC = 1.30).

A pairwise comparison of fold change EC_50_ values among all patient-derived T97A viruses indicated a strong correlation between EVG and RAL susceptibilities (r^2^ = 0.74; *P* < 0.001) (Fig 6). Strong correlations were also observed among isolates with pre-existing T97A (r^2^ = 0.82; *P* < 0.001) and isolates with emergent T97A alone (r^2^ = 0.87; *P* = 0.002) (**data not shown**). However, only 36% (16 of 45) of patient-derived T97A viruses with data exhibited reduced susceptibility to both EVG and RAL greater than both biological cutoffs. The 2 TE patients with the highest EVG FC values (9.94 and 8.95) developed T97A on RAL+ABC+LPV/r and co-developed the secondary INSTI RAM, L74M (or L74L/M). When removed from the data set, the fold change difference in EVG susceptibility between pre-existing and emergent T97A patient populations was less significant than reported above (mean FC = 2.94 vs. 4.71 to 2.94 vs. 2.81; *P* = 0.077 to 0.897).

### Impact of T97A alone (pre-existing or emergent) on INSTI-based treatment outcome and resistance development

Among the 17 patients with pre-existing T97A and available clinical follow-up (17 of 18), 16 patients [15 TN patients on EVG/COBI/FTC/TDF and 1 TE patient on EVG/r+TDF+DRV] achieved and maintained high rates of virologic suppression (16 of 17 with data; 94%) and 1 patient (described below) experienced virologic rebound (1 of 17 with data; 6%). One patient [1 TE patient on EVG/r+TDF+DRV] did not achieve virologic suppression prior to early study drug discontinuation at a non-protocol-defined virologic failure visit (Week 8) and was excluded from treatment outcome analysis, consistent with previously reported study-specific efficacy analyses (see Methods) (Fig 2; S1 Table and S1 Fig). The proportion of patients with pre-existing T97A and successful INSTI-treatment outcome (16 of 17 with data; 94%) was similar to the overall treatment population (3759 of 3881; 97%)(*P* = 0.42). The 1 patient [1 TE patient on EVG/r+TDF+DRV] that experienced confirmed viral rebound (HIV-1 RNA = 38,900 copies/mL) at a first protocol-defined virologic failure visit (Week 40) on EVG/r+TDF+DRV maintained T97A and sensitivity to EVG without further development of antiretroviral resistance. This patient had pre-existing NNRTI (V90I) resistance, was noncompliant (overall adherence rate = 84%), and discontinued study drug 21 days later (HIV-1 RNA = 330 copies/mL). Overall, pre-existence of T97A at screening or baseline (with or without reduced INSTI susceptibility) does not appear to affect treatment response to INSTI-based therapy with two other fully active antiretrovirals.

Among the 7 patients with emergent T97A alone (i.e., in the absence of primary INSTI RAMs) at virologic failure and available clinical follow-up (7 of 8), 1 patient [1 TN patient on EVG/COBI/FTC/TDF] maintained M184V in reverse transcriptase and T97A in integrase before regaining virologic suppression with no change in therapy (1 of 7 with data; 14%) and 6 TE patients remained viremic (6 of 7 with data; 86%) [25, 89]. One TE patient withdrew consent and discontinued study drug at Week 144 and was excluded from treatment outcome analysis, consistent with previously reported study-specific efficacy analyses (see Methods) (Fig 2; S3 Table and S3 Fig). Of the 6 TE patients that remained viremic: (i) 2 patients did not meet protocol-defined virologic failure criteria for repeat resistance testing, (ii) 1 patient lost T97A, (iii) 2 patients maintained T97A for 10 and 73 weeks, respectively, without a further significant reduction in INSTI susceptibility, and (iv) 1 patient developed T97T/A at Week 12 and then switched primary INSTI resistance pathways to T66T/A and S147S/G at Week 20 before discontinuing study drug 4 weeks later.

## Discussion

The introduction of INSTI-based regimens for the treatment of HIV-1 infection has proven highly effective and well-tolerated in INSTI-naive patients with or without prior antiretroviral experience. Nevertheless, major/primary INSTI RAMs may develop at highly conserved integrase positions in patients experiencing virologic failure on INSTI-based therapy. Although all major/primary INSTI RAMs that significantly reduce INSTI susceptibility are treatment-selected mutations, the converse is not necessarily true [58]. Many minor/secondary INSTI RAMs (including naturally occurring integrase polymorphisms) do not compromise INSTI activity on their own [50, 64, 65], yet frequently arise in INSTI-treated patients [51, 62, 66, 79, 106]. The T97A integrase mutation, considered a secondary INSTI RAM in most publications, has been identified periodically in patients experiencing virologic failure on INSTI-based therapy [25, 66, 80] and is also an infrequent integrase polymorphism associated with low-level reduced susceptibility to EVG and/or RAL [45, 50, 86, 87]. However, little is known about the impact of T97A on subsequent therapy with EVG- or RAL-containing regimens. Here, we performed a cross-study pooled analysis of the impact of the T97A integrase mutation (pre-existing and emergent) on INSTI susceptibility and treatment outcome to provide context and guidance on the need for integrase genotyping before INSTI therapy.

To date, most reports show that primary INSTI RAMs are not present prior to the initiation of INSTI therapy [50, 52–65, 107–109]. Similarly, our analysis of 3367 integrase sequences derived from TN or TE patients (all INSTI-naive by definition) showed that no primary INSTI RAMs (excluding T97A) and very few secondary INSTI RAMs were present prior to initiation of INSTI-based therapy. Since natural genetic variability has been observed in the integrase gene [51–53, 58, 63, 64], it was not unexpected that some secondary INSTI RAMs would be detected at low to moderate frequencies. Taken together, these data confirm that transmitted INSTI resistance is rare through year 2013 (last year of study enrollment) and that moderate-to-high level INSTI resistance does not spontaneously occur among INSTI-naive patients.

In agreement with previous studies [52, 53, 56, 58, 63, 64, 110], the T97A integrase mutation was identified as a naturally occurring low frequency integrase polymorphism among INSTI-naive patients (47 of 3367; 1.4%) and confirmed among a much larger data set of available integrase sequences (255 of 13455; 1.9%). Since T97A can occur before or during INSTI-based treatment, HLA-association of this integrase polymorphism [111] may impart a selective advantage to evade cellular immune response which could impact frequency of transmitted INSTI resistance. However, further investigation is needed to better assess the clinical impact and prevalence of T97A as an escape mutation at specific epitopes relative to corresponding HLA alleles in different patient populations. In agreement with previous reports in RAL-treated patients [61, 112], few patients experienced virologic failure with emergent T97A on EVG or RAL-based therapy (14 of 122; 11%). Detection of T97A in the presence of a primary INSTI RAM (n = 6) was consistent with previous observations of further reduced INSTI susceptibility and increased viral fitness relative to each primary INSTI RAM [47, 83, 84]. By comparison, emergent T97A alone (i.e., in the absence of primary INSTI RAMs) was inconsistently associated with reduced EVG and/or RAL susceptibility, similar to isolates with pre-existing T97A. However, the selective advantage of emergent T97A during continued INSTI-based therapy remains unclear due to the low number of patients with clinical follow-up data and their varied outcomes.

Because genetic barrier is an important determinant of drug resistance, genetic variability among patients with different HIV-1 subtypes may influence the ease with which an integrase mutation can occur before or during INSTI treatment [44, 52, 56, 58, 60, 61, 66–70, 113]. Previous studies have indicated that the prevalence of specific integrase polymorphisms, characterized as emergent secondary INSTI RAMs in subtype B, are greater among patients with non-B subtypes [56–58, 60, 61, 110] and therefore may impact the magnitude of INSTI resistance, virologic response, or the propensity to acquire primary INSTI RAMs [68]. Similar to earlier reports, we found that T97A pre-existed more frequently among patients harboring non-B than B subtypes [53, 56, 58, 59, 61]. An analysis of a much larger data set further indicated that the low prevalence of T97A rarely differs by more than 10-fold between subtypes. Since these differences could not be explained by differences in codon usage at position 97, it is possible that they may instead reflect subtype-specific sequence differences. Overall, T97A did not emerge more frequently in patients harboring non-B subtypes, however, generation of T97A as a single nucleotide transition polymorphism implies that this genetic change can occur easily among most subtypes.

Because HIV-1 explores sequence space under drug pressure by selecting resistance and compensatory mutations, adaptive evolution in treatment-experienced patients could also shape integrase variability before or during INSTI treatment. Indeed, prior antiretroviral exposure has been associated with greater integrase divergence, leading to the suggestion that treatment-induced changes in other parts of the HIV-1 genome may be linked to the prevalence of specific integrase polymorphisms [51, 54, 56, 59, 64, 70, 81, 114]. In our analysis, pre-existing T97A was detected less frequently among INSTI-naive patients with prior antiretroviral exposure (TE: 3 of 703; 0.4%) than without (TN: 44 of 2664; 1.7%) and prior antiretroviral exposure was not specifically associated with reduced INSTI susceptibility. In contrast, a previous study reported a higher frequency of pre-existing T97A among INSTI-naive patients with prior antiretroviral exposure (TE: 4 of 166; 2.4%) than without (TN: 0 of 249; 0%) [56]. Thus, the clinical significance of T97A frequencies among INSTI-naive patients could be attributable to multiple factors, including, but not limited to enrollment bias over time or differences among prior antiretroviral regimens. During INSTI-based therapy, however, T97A emerged more frequently among TE than TN patients, consistent with previous studies of primary INSTI RAMs [22, 25, 51]. Nevertheless, the fact that most TE and TN patients with pre-existing or emergent T97A retained full or partial susceptibility to EVG and RAL and full susceptibility to DTG should be considered reassuring in contemporary clinical practice.

An important tool in the prognosis of HIV-1 drug resistance and impact on treatment-outcome is the prediction of phenotypic INSTI sensitivity based on genotypic information using virtual software-based algorithms. However, there exist varying degrees of discordance in these interpretative algorithms of the level of INSTI sensitivity conferred by integrase mutation(s). While ANRS [115] and REGA [116] consider genotype-outcome correlations, HIVdb [117] balances genotype-outcome, genotype-phenotype, and genotype-treatment correlations, and GeneSeq/GenoSure Integrase [88] uses genotype-phenotype correlations. Of the INSTI RAMs detected as naturally occurring integrase polymorphisms, only L74M, T97A, E157Q, and G163R are considered secondary INSTI RAMs by at least one algorithm. Of interest, the T97A integrase mutation is considered susceptible to INSTIs by ANRS and REGA algorithms, while the level of resistance reported by other algorithms differs: HIVdb (EVG: potential low-level; RAL: low-level) and GeneSeq/GenoSure integrase (EVG: reduced susceptibility/resistant possible; RAL: susceptible).

Our study provides useful information concerning the phenotypic INSTI sensitivity of viral isolates harboring T97A relative to treatment outcome. Differences in susceptibilities to EVG and/or RAL were noted among T97A-containing isolates relative to their biological cutoffs using the PhenoSense^™^ Integrase assay, ranging from low-level to no effect at all. In contrast, a site-directed T97A mutant virus was previously shown to exhibit low-level reduced EVG susceptibility and no effect on RAL susceptibility [45]. Because many mutations can act additively or synergistically to reduce drug susceptibility [87, 117, 118], these results suggest that inter-patient genetic integrase diversity may impact T97A-based INSTI resistance. Indeed, additional secondary INSTI RAMs (e.g., L74M or G163R) may associate with T97A to further reduce susceptibility to EVG and/or RAL in the absence of primary INSTI RAMs [86–88], however the selection of these combinations during INSTI-based therapy appears infrequent.

In the current study, the T97A+L74M combination was detected in 2 TN patients with pre-existing T97A+L74M and 2 TE patients with emergent T97A+L74M (S1 and S3 Tables). Although phenotypic data was available for only the 2 TE patients with emergent T97A+L74M, this combination exhibited the largest reduction in INSTI susceptibility among all T97A-containing isolates, albeit less than 10-fold. Other integrase mutations and/or secondary INSTI RAM(s) (i.e., M50I, V72N, S119G/P/R, and G163R), identified in INSTI-naive patients with or without pre-existing T97A, also persisted or emerged in patients that developed T97A alone. In particular, a striking number of patients who developed T97A also had S119 polymorphisms. Thus, further study is needed to resolve the phenotypic significance of integrase polymorphism(s) that may associate with T97A. Nonetheless, our data collectively agree with the predictions of ANRS and REGA in that pre-existing T97A with or without phenotypic INSTI resistance does not significantly impact treatment outcome. This is important as it suggests that maintenance of reasonable systemic exposures of pharmacoenhancer-boosted EVG (85/125/150 mg once a day) or RAL (400 mg twice a day) as part of a complete antiretroviral regimen are sufficient to inhibit the T97A integrase mutation [119, 120]. Henceforth, periodic surveillance of T97A integrase mutation patterns and correlation with phenotypic INSTI susceptibility may be necessary to sustain the accuracy of EVG and RAL genotypic algorithms relative to biological or clinical thresholds.

Since T97A has been associated with INSTI resistance in patients experiencing virologic failure on EVG or RAL [25, 85–89], we sought to address the concern of whether this naturally occurring integrase polymorphism could change the current paradigm for supplemental integrase genotyping prior to INSTI-based therapy. Our analyses showed that only 1 of 18 patients with pre-existing T97A who enrolled on INSTI-based therapy experienced virologic failure. Importantly, in this single occurrence of persistent T97A (no change from pre-treatment sequence), detection at virologic failure was not associated with reduced INSTI susceptibility in vitro. These findings suggest that pre-existence of T97A, with or without partially reduced INSTI susceptibility, does not significantly impact treatment outcome or impart a strong selective advantage under INSTI pressure to drive virus escape and/or the evolution of INSTI resistance. Although our analyses were limited to mostly patients enrolled on an EVG-based regimen, other reports have suggested that pre-treatment presence of T97A does not predispose poor treatment outcome or resistance to RAL or DTG as well [22, 66, 86, 109, 114]. These results are also consistent with previously studied integrase polymorphisms, including some minor/secondary INSTI RAMs (e.g., S119P and G163R) [22, 23, 56, 66, 114, 121], but discordant from major/primary INSTI RAMs [60, 122]. Therefore, pre-existence of T97A, with or without additional integrase mutations, poses a minimal risk of compromised virologic response for INSTI-naive patients enrolling on INSTI-based therapy.

## Conclusions

Overall, natural or transmitted INSTI resistance in INSTI-naive HIV-1 infected patients was extremely rare, confined to only a restricted few integrase polymorphisms. The T97A integrase mutation, infrequently associated with emergent EVG and RAL resistance at virologic failure, was identified as a low frequency naturally occurring integrase polymorphism of greater prevalence in HIV-1 non-B than B subtypes. While T97A may confer low-level reductions in INSTI susceptibility in the context of other integrase polymorphisms, mutational pathways of INSTI resistance at virologic failure were not driven by the persistence of T97A. More importantly, pre-treatment detection of T97A did not increase the risk of virologic failure on EVG or RAL-based therapies. Taken together, these analyses support the current clinical paradigm that INSTI-naive patients are likely to benefit from INSTI-based therapy without a concern of inherent reduced susceptibility or compromised virologic response based on natural integrase polymorphisms.

## Supporting information

S1 Table Pre-Treatment Population of Patients with Pre-Existing T97A (n = 47). (PDF)

S2 Table On-Treatment Population of Patients with Emergent T97A and Primary INSTI RAM(s) (n = 6). (PDF)

S3 Table On-Treatment Population of Patients with Emergent T97A Alone (n = 8). (PDF)

S1 Fig Pre-Treatment Population of Patients with Pre-Existing T97A (n = 47): Longitudinal Plots. (PDF)

S2 Fig On-Treatment Population of Patients with Emergent T97A and Primary INSTI RAM(s) (n = 6): Longitudinal Plots. (PDF)

S3 Fig On-Treatment Population of Patients with Emergent T97A Alone (n = 8): Longitudinal Plots. (PDF)
