# Supplementary material for: Lack of impact of pre-existing T97A HIV-1 integrase mutation on integrase strand transfer inhibitor resistance and treatment outcome
Source: PLoS One. 2017 Feb 17;12(2):e0172206. doi: 10.1371/journal.pone.0172206 (PMC5315389; doi:10.1371/journal.pone.0172206)
Supplement: S1 Table — (PDF) [file pone.0172206.s001.pdf]

S1 Table. Pre-Treatment Population of Patients with Pre-Existing T97A (n = 47)

PID #	Study #	Treatment Group	INSTI Dose	TE/ TN	HIV-1 Subtype	Snapshot Outcome	Drug Completion Reason	Adhr Rate (%)[a]	RAP[b]	Visit	HIV-1 RNA	CD4	Integrase Sequence[c, d]	INSTI RAM(s)[d]	T97A Status	IN Phenotypic Data[e]				RTI RAM(s)[f]	PI RAM(s)[g]
																RC	EVG FC	RAL FC	DTG FC		
**Enrolled on INSTI-based Regimen**																					
1	GS-US-183-0145	EVG/r+TDF+DRV	150 mg	TE	B	VS (W96)	ND	86	-	SCR	13900	434	S24S/G S39C L45V M50I I84L P90P/S T97T/A V113I T124T/A V201I V234L	T97T/A M50I		ND	1.18	0.82	0.94		I13V L63P
2	GS-US-236-0102	EVG/COBI/FTC/TDF	150 mg	TN	B	VS (W144)	ND	91	-	BL	103000	1311	V31I T97A K111T T124A T206S V234L	T97A		ND	2.75	1.58	ND		M36I L63P A71V V77I I93L
3	GS-US-236-0102	EVG/COBI/FTC/TDF	150 mg	TN	B	VS (W144)	ND	98	-	BL	22900	396	D6E K14R S17N S39C M50I K71Q V72I S81S/R T97T/A L101I V113I S119R T125A V201I V234L I268L	T97T/A M50I V72I S119R		ND	1.97	1.65	ND		L10V I13V L63P
4	GS-US-236-0102	EVG/COBI/FTC/TDF	150 mg	TN	B	VS (W144)	ND	99	-	BL	13900	344	S17N V31I Q53H V72I P90P/S T97T/A L101L/I L104L/I T124N T125A G163A V201I K211Q I220V V234L V260I	T97T/A V72I G163A		ND	1.87	1.34	ND		
5	GS-US-236-0103	EVG/COBI/FTC/TDF	150 mg	TN	B	VS (W144)	ND	100	-	SCR	109000	301	E11D V31I S39N V72I A91E T97T/A Y99Y/F L101I K111N V113I S119R T124N T125T/A I135V V201I K219N N222K S230N D232E V234L	T97T/A V72I S119R		ND	2.15	1.30	0.87	K103K/N	L63P/Q I64I/V
6	GS-US-236-0103	EVG/COBI/FTC/TDF	150 mg	TN	B	VS (W144)	ND	100	-	SCR	16200	466	K7K/R S39C M50I V72I T97A V113I S119S/R T124A T125A K211Q D232D/E V234L	T97A M50I V72I S119S/R		ND	7.53	2.25	0.84		M36I I62V L63P V77I I93L
7	GS-US-236-0128	EVG/COBI/FTC/TDF	150 mg	TN	D	VS (W48)	ND	94	-	SCR	7910	507	S17N L28I L45L/I V79V/I T97A T112V V113V/I S119R T125A D167E K173R V201I I208I/M V234I K240K/R D256E A265V D286N	T97A S119R		73	2.85	1.18	0.67		I13V M36I I64V
8	GS-US-236-0128	EVG/COBI/FTC/TDF	150 mg	TN	A	VS (W48)	ND	98	-	SCR	119000	637	E11E/K V31I D41D/N I60M T97A T112V T124A T125A V126F G134N I135V K136Q F139Y D167E V201I T218T/I I220I/L Q221Q/H V234I/L A265A/V R269R/K D278D/A S283G	T97A		44	3.32	2.24	0.45		I13V M36I H69K
9	GS-US-236-0128	EVG/COBI/FTC/TDF	150 mg	TN	A1	VS (W48)	ND	99	-	BL	4950	287	S17N D25E V31I S39S/N L63I L74I I84L T97A S119T T124A T125A V126F G134N K136Q P142P/S D167E V201I T218L V234I S255N	T97A L74I S119T		84	2.97	2.22	0.49		L10V I13I/V G16E M36I/V I62V H69K
10	GS-US-236-0128	EVG/COBI/FTC/TDF	150 mg	TN	A	VS (W48)	ND	100	-	BL	366	699	K14R A21T V31I T97A T112V T124A T125A V126F G134N I135V K136Q D167E K173R V201I V234I S283G	T97A		207	4.20	1.55	0.56	V118I	I13V M36I I62V H69K
11	GS-US-236-0128	EVG/COBI/FTC/TDF	150 mg	TN	D	VS (W48)	ND	95	-	BL	143000	577	S17N L28I S39C M50L V72I T97T/A L101I T112M V113I S119S/G/R T122T/I T124A T125A V201I T218I V234I S283G	T97T/A M50L V72I S119S/G/R		ND	2.76	1.52	1.25	K101E G190A	I13V M36I D60E I64V
12	GS-US-236-0128	EVG/COBI/FTC/TDF	150 mg	TN	A1	VS (W48)	ND	100	-	SCR	45600	290	S17N I60M T97A L101I V110V/I T124A T125A V126F G134N D167E G193D V201I T210T/I K211R T218I V234I R269K D270H S283G	T97A		1	2.22	1.33	0.58		I13V M36I H69K
13	GS-US-236-0128	EVG/COBI/FTC/TDF	150 mg	TN	A1	VS (W48)	ND	100	-	SCR	11000	590	K14R S24G V32I S39N L45Q L74I T97A T112I V113A T124A T125A V126F G134D I135V D167E V201I K211R N222K V234I S283G	T97A L74I		20	2.33	0.99	0.54	K103N E138G V179T	I13V M36I/L H69K
14	GS-US-236-0128	EVG/COBI/FTC/TDF	150 mg	TN	A1	VS (W48)	ND	94	-	SCR	22100	497	A21A/S V32I D41D/N I60M T97A T112V T124A T125A V126F G134S K136Q D167E V201I T218T/I V234I D256D/E S283G	T97A		39	1.94	1.23	0.57		L10V I13V K20R M36I H69K
15	GS-US-236-0128	EVG/COBI/FTC/TDF	150 mg	TN	G	VS (W48)	ND	96	-	BL	29500	244	K14R S17N/T V31I V72I T97T/A L101I T112I V113I H114H/Y T124A T125A G134N K136T V201I T206S V234I S255N D256E S283G	T97T/A V72I		68	1.33	1.21	1.04		I13V K20I M36I L63L/P H69K V82I
16	GS-US-292-0111	EVG/COBI/FTC/TDF	150 mg	TN	B	VS (W96)	ND	47%	-	SCR	9290	422	E10D K14K/R V31I K71Q V72I I84M T97T/A L101I V113I S119R K188K/R V234I/L	T97T/A V72I S119R		41	4.19	1.49	0.77		M36I L63P A71A/T
17	GS-US-183-0145	EVG/r+TDF+DRV	150 mg	TE	B	VF (W96)	Withdrew Consent	47%	-	SCR	8880	27	S17N S39C T97A L101I V113I S119T T124G T125A K156N V201I V234L	T97A S119T		ND	2.61	0.64	ND	T69D/N M184M/V K219R K101E G190S	G16E M36I I62V L63P
	GS-US-183-0145	EVG/r+TDF+DRV	150 mg	TE	B	VF (W96)	Withdrew Consent	47%	-	ESDD (W8)	8970	27	ND	ND		ND	ND	ND	ND	ND	ND
18	GS-US-183-0145	EVG/r+TDF+DRV	150 mg	TE	G	VF (W96)	Subject Non-Compliance	84%	W48	BL	66100	340	K14R R20K V31I V32V/A A33A/T V72I T97A L101I T112I/V V113I T124A T125A G134N I135V G163Q G193E V201I T206S T218T/I Y227F V234I S255N D256E D279G S283G	T97A V72I G163Q		ND	1.84	1.14	ND	V90I	I13V K20I M36I H69K V82I
	GS-US-183-0145	EVG/r+TDF+DRV	150 mg	TE	G	VF (W96)	Subject Non-Compliance	84%	W48	W40	38900	ND	K14R R20K V31I V72I T97A L101I T112I/V V113I T124A T125A G134N I135V G163Q G193E V201I T206S T218T/I Y227Y/F V234I S255N D256E D279G S283G	T97A V72I G163Q	Maintained	ND	2.19	1.3	ND	V90I	I13V K20I M36I H69K V82I
	GS-US-183-0145	EVG/r+TDF+DRV	150 mg	TE	G	VF (W96)	Subject Non-Compliance	84%	W48	ESDD (W48)	330	497	ND	ND	Not tested again	ND	ND	ND	ND	ND	ND

PID #	Study #	Treatment Group	INSTI Dose	TE/ TN	HIV-1 Subtype	Snapshot Outcome	Drug Completion Reason	Adhr Rate (%)[a]	RAP[b]	Visit	HIV-1 RNA	CD4	Integrase Sequence[c, d]	INSTI RAM(s)[d]	T97A Status	IN Phenotypic Data[e]				RTI RAM(s)[f]	PI RAM(s)[g]
																RC	EVG FC	RAL FC	DTG FC		
**Not Enrolled or Not Enrolled on INSTI-based Regimen**																					
**19**	GS-US-99-903	d4T+3TC+EFV	-	TN	A	ND	ND	ND	-	BL	5094	624	K7K/R V72I L74L/I T97T/A L101I T112V V113I T124A T125A G134N K136Q V201I R231R/K V234I S283G	T97T/A V72I L74L/I		ND	1.68	1.08	0.72		I13V K20I M36I H69K
**20**	GS-US-236-0102	EFV/FTC/TDF	-	TN	B	VF (W144)	Lost To Follow-Up	100%	W96	BL	11900	512	E11D V31I M50M/T G70E V72I T97A L101I T112I V113I S119R T124N D167E V201I T206S I208L E212A T218S V234L K240K/R S255N D256E R284G	T97A M50M/T V72I S119R		177	6.08	2.59	ND		M36I
**21**	GS-US-236-0103	ATV/r+FTC/TDF	-	TN	B	ND (W144)	Lost To Follow-Up	96%	W48	BL	238000	208	S24G S39C V79A T97T/A L101I V113I T124N V201I Q216R K219Q V234L A265V S283G	T97T/A		ND	1.00	0.80	ND	V118I	K20R I62V L63P V77I I93L
**22**	GS-US-236-0128	ATV/r+FTC/TDF	-	TN	A1	VF (W48)	Lack Of Efficacy	99%	W48	BL	52200	345	R20K V31I L74I V77V/A T97T/A L101L/I T112V V113I S119P T124S T125A G134N K136Q D167E V201I T206T/S D207D/E I208I/L T210T/I T218I V234I S255N	T97T/A L74I S119P		AF	AF	AF	AF	K103N	I13V M36I H69K I93I/L
**23**	GS-US-292-0104	-	-	TN	B	-			-	SCR	22400	ND	E11D R20R/K V31I G70E V72I V75V/M T97A L101I T112V V113I S119R T124N/S T125T/A I135V F181F/L V201I T206S V234L V281M	T97A V72I S119R		124	10.70	4.04	0.66		M36M/I I62V L63P/S I64I/L/V
**24**	GS-US-292-0104	-	-	TN	B	-			-	SCR	24900	ND	E11D V72I A91E T97A L101I K111Q V113I S119R V201I V234L S283G	T97A V72I S119R		134	6.78	3.04	0.79	V90V/I	L63P A71V V77V/I I93L
**25**	GS-US-292-0104	-	-	TN	B	-			-	SCR	14700	ND	V31I M50I G70E T97A L101I V113I S119R T124A T125A I135V F181F/L V201I V234L D256E	T97A M50I S119R		163	6.21	2.37	0.68		G16E D60E I62V I64V V77I
**26**	GS-US-292-0104	-	-	TN	AG	-			-	SCR	4790	ND	E11D S24N V31I V72I T97T/A L101I T112V V113I S119T T124A T125A G134N I135V K136T E157Q K160Q V201I I203M T206S I208L T218I V234I A265V S283G R284G D288N	T97T/A V72I S119T E157Q		107	2.62	1.58	0.94		I13V K20I M36I H69K
**27**	GS-US-292-0104	-	-	TN	B	-			-	SCR	30500	ND	E11D S17T A23V V72V/I T97A L101I K111A/T V113I T124A V234L	T97A V72V/I		112	2.20	1.41	0.65		I62I/V V77V/I
**28**	GS-US-292-0104	-	-	TN	B	-			-	SCR	6920	ND	S17N V31I T97A L101I T112I V113I T124A K136Q V234L D253E D256E	T97A		144	1.92	1.41	0.97	K103N Y181C H221Y	M46L Q58E V82A L10I K20R M36I I54V I62V I64V A71T
**29**	GS-US-292-0104	-	-	TN	B	-			-	SCR	26500	ND	S17N V31I T97A L101I V113I T124A Q177V K211R Y227F V234L K264R A265V D288N	T97A		44	1.64	1.30	0.52		L63P V77I I93L
**30**	GS-US-292-0104	-	-	TN	B	-			-	SCR	3330	ND	E10A S17N/T V31I V72I T97A L101I R107R/G T112T/I V113I T124A D167E G193E V234L D256E D278A S283G	T97A V72I		5	1.57	1.20	0.64	V90V/I	M36I I64V
**31**	GS-US-292-0104	-	-	TN	AG	-			-	SCR	6850	ND	E11D K14R R20R/K S24N V31I I60I/T G70G/E V72I E96E/D T97T/A L101I K103R T112V V113I T124A T125A G134N I135I/V K136T D167D/E V201I T206S V234I V259I R269K S283G D286N	T97T/A V72I		59	1.39	1.06	0.61		I13V K20I E35G M36V I64I/M H69K V77I
**32**	GS-US-292-0104	-	-	TN	B	-			-	SCR	107000	ND	V32I M50I I84M E96E/D T97T/A L101I L104L/I V113I T124A G193E V201I T206S T218A/S N222H V234L D253E S255N D256E D279G	T97T/A M50I		97	1.14	0.90	0.9	T69N	I62V L63P I93L
**33**	GS-US-292-0104	-	-	TN	A1	-			-	SCR	15200	ND	K14R V31I L45Q L74M T97A L101V T112V V113I T124A T125A V126F G134N I135V K136Q D167E K173R V201I T210T/I T218T/S V234I D279G S283G	T97A L74M		AF	AF	AF	AF		I13V M36I I62I/V H69K
**34**	GS-US-292-0104	-	-	TN	B	-			-	SCR	3550	ND	E11D S17N A21S A23V V72I T97A L101I K103K/R T112T/I V113I S119P T122I T125A V234L A265A/V	T97A V72I S119P		ND	4.3	1.39	0.71		M36I L63P A71T
**35**	GS-US-292-0104	-	-	TN	AE	-			-	SCR	30100	ND	K14R A21T V31I L74M H78R T97A T112I V113I T124A T125A G134N I135V K136R D167E V201I I203M T206S V234I S283G	T97A L74M		AF	AF	AF	AF		I13V M36I H69K
**36**	GS-US-292-0106	-	-	TN	Complex	-			-	SCR	25000	ND	K14K/R S17N A23A/V S24S/G V31V/I V32V/I S39C M50I T93T/K/N T97T/A L101I T112V T124A T125A K136Q D167E V201I A205A/S T206S E212E/A V234L D286D/N	T97T/A M50I		ND	ND	ND	ND	E138A	G16E K20K/T V77V/I
**37**	GS-US-292-0106	-	-	TN	D	-			-	SCR	131000	ND	S17N M50L T97T/A L101L/I T112V S119P/T T124A T125A V201I T206S I208I/L T210T/I Q214Q/E T218I V234L D256E S283G	T97T/A M50L S119P/T		ND	ND	ND	ND		I13I/V M36I I62V I64V
**38**	GS-US-292-0106	-	-	TN	D	-			-	SCR	63700	ND	S17N M50L K71Q V72I T97A L101I T112V S119R T122T/A T125A V201I D256E A265V R269R/K	T97A M50L V72I S119R		ND	ND	ND	ND		I13V L33V I64V
**39**	GS-US-292-0106	-	-	TN	A1	-			-	SCR	44000	ND	K14R A21T V31I I60I/M I84L T97A T124A T125A V126F G134N I135I/V K136Q D167E K188K/R G193G/E V201I I208M V234I S255S/N A265V R269R/K D278D/A D286N	T97A		ND	ND	ND	ND		I13V M36I H69K

PID #	Study #	Treatment Group	INSTI Dose	TE/ TN	HIV-1 Subtype	Snapshot Outcome	Drug Completion Reason	Adhr Rate (%)[a]	RAP[b]	Visit	HIV-1 RNA	CD4	Integrase Sequence[c, d]	INSTI RAM(s)[d]	T97A Status	IN Phenotypic Data[e]				RTI RAM(s)[f]	PI RAM(s)[g]
																RC	EVG FC	RAL FC	DTG FC		
**40**	GS-US-292-0111	-	-	TN	B	-			-	SCR	51200	ND	E11D S17T A23V V37I V72I T97A L101I V113I T124A T125A I135V V201I V234L D256E	T97A V72I		79	2.38	1.27	0.73		L10V I64L
**41**	GS-US-292-0111	-	-	TN	B	-			-	SCR	4770	ND	E11D A21T A23V K34K/R L45V M50I V72I T93S T97T/A L101L/I V113I T124A V165I S195T V201I Q216H V234L S255R	T97T/A M50I V72I		84	1.76	1.30	1.04	E138A	L63L/P I64V V77I
**42**	GS-US-292-0111	-	-	TN	B	-			-	SCR	4650	ND	S17S/N V31I V72I T97T/A L101L/I V113I T124A I182V K219N N222K V234L D256E	T97T/A V72I		122	1.51	1.08	0.94		D60E L63P A71T V77I
**43**	GS-US-292-0111	-	-	TN	B	-			-	SCR	176000	ND	V31I L45V V72I T97T/A L101I T112V V113I T124Q T125A I135V V201I V234L	T97T/A V72I		110	1.25	1.08	0.95	M41L	L33F M46L L76V L10I I13V L24I M36L I54V I62I/V L63P V82I I85V
**44**	GS-US-292-0111	-	-	TN	B	-			-	SCR	35900	ND	S17N T97T/A L101I T124A T125A I161I/T G163E/K T206S V234L V281M	T97T/A G163E/K		44	1.10	0.98	0.87	V118I K103N E138G Y188L	L63P
**45**	GS-US-292-0111	-	-	TN	A1	-			-	SCR	12900	ND	K14R S24N V31I I60M T97T/A K103K/R T112V T124A T125A V126F G134N K136Q F139Y D167E Q177L V201I I203M K211R K219N N222K V234I S255N A265V S283G D286N	T97T/A		AF	AF	AF	AF	V179I/T	I13V G16E M36I H69K
**46**	GS-US-292-0111	-	-	TN	B	-			-	SCR	10200	ND	D25E V31I V72I Q95Q/P E96E/D T97T/A L101I K111R V113I D167E V201I T206S I220L V234L R262K I268L	T97T/A V72I		AF	AF	AF	AF		V77I
**47**	GS-US-292-0111	-	-	TN	A1	-			-	SCR	83400	ND	S17N M50I K71Q L74I T97A T112V V113I S119R T124A T125A K136Q D167E K173K/R V201I T218S Y227F V234V/I S283G	T97A M50I L74I S119R		ND	4.15	1.81	0.84		I13V M36I H69K

[a] Overall adherence rate as determined by pill count in returned pill bottles.

[b] The resistance analysis population (RAP) included subjects with either 1) virologic rebound (VR): HIV-1 RNA ≥50 copies/mL at any visit , that is confirmed and ≥400 copies/mL at the next scheduled or unscheduled visit; or 2) Discontinuation from study with HIV-1 RNA ≥400 copies/mL at last visit (Last) while receiving study drugs (or within 72 hours of discontinuation). Subjects who were not receiving study drugs within 72 hours prior to virologic rebound (Off SD) were not analyzed for resistance.

[c] Monogram Biosciences (South San Francisco, CA) GeneSeq™ Integrase or GenoSure™ Integrase assay

[d] Post-baseline IN sequence was compared to Baseline (BL) or Screening (SCR) IN sequence with emergent RAMs shown in **bold**. Primary INSTI RAMs are: T66A/I/K, E92G/Q, T97A, Y143C/H/R, S147G, Q148H/K/R, and N155H. Secondary INSTI RAMs are: M50I, H51Y, L68I/V, V72A/N/T, L74I/M, Q95K/R, G118R, S119G/P/R/T, F121C/Y, A128T, E138A/K, G140A/C/S, P145S, Q146I/K/L/P/R, V151A/L, S153A/F/Y, E157K/Q, G163K/R, E170A, and R263K. Other variants at IN positions associated with INSTI RAMs are also indicated.

[e] Monogram Biosciences (South San Francisco, CA) PhenoSense™ Integrase assay. Replication capacity (RC) expressed as % of wild-type control. Drug susceptibility data expressed as EC_{50} fold change (FC) from wild-type control.

[f] NRTI mutations are: M41L, E44D, A62V, K65R, D67N, T69D/N, T69 insertion, K70E/R, L74I/V, V75I, F77L, Y115F, F116Y, V118I, Q151M, M184I/V, L210W, T215F/Y, and K219E/N/Q/R in RT. NNRTI-R mutations are: V90I, A98G, L100H/I, K101E/P, K103N/S, V106A/I/M, V108I, E138A/G/K/Q/R, V179D/F/L/T, Y181C/I/V, Y188L/C/H, G190A/E/Q/S, H221Y, P225H, F227C, M230I/L

[g] Primary PI-R mutations are: D30N, V32I, L33F, M46I/L, I47A/V, G48V, I50L/V, I54L/M, Q58E, T74P, L76V, V82A/F/L/S/T, I84V, N88S, and L90M in PR. Secondary PI-R mutations are: L10C/F/I/R/V, V11I, I13V, G16E, K20I/M/R/T/V, L24I, L33I/V, E34Q, E35G, M36I/L/V, K43T, F53L/Y, I54A/S/T/V, D60E, I62V, L63P, I64L/M/V, H69K, A71I/L/T/V, G73A/C/S/T, V77I, V82I, N83D, I85V, N88D, L89V, and I93L/M in PR.

AF: assay failure; DTG: dolutegravir; EVG: elvitegravir; IN: integrase; INSTI: integrase strand transfer inhibitor; ND: no data; PI: protease inhibitor; PID: patient identification; RAL: raltegravir; RAM: resistance-associated mutation; RTI: reverse transcriptase inhibitor; VF: virologic failure; VS: virologic success.
