# Supplementary material for: Lack of impact of pre-existing T97A HIV-1 integrase mutation on integrase strand transfer inhibitor resistance and treatment outcome
Source: PLoS One. 2017 Feb 17;12(2):e0172206. doi: 10.1371/journal.pone.0172206 (PMC5315389; doi:10.1371/journal.pone.0172206)
Supplement: S2 Table — (PDF) [file pone.0172206.s002.pdf]

S2 Table. On-Treatment Population of Patients with Emergent T97A and Primary INSTI RAM(s) (n = 6)

PID #	Study #	Treatment Group	INSTI Dose	TE/ TN	HIV-1 Subtype	Snapshot Outcome	Drug Completion Reason	Adhr Rate[a]	RAP[b]	Visit	HIV-1 RNA	CD4	Integrase Sequence[c, d]	INSTI RAM(s)[d]	T97A Status	IN Phenotypic Data[e]				RTI RAM(s)[f]	PI RAM(s)[g]
																RC	EVG FC	RAL FC	DTG FC		
**Enrolled on INSTI-based Regimen**																					
**48**	GS-US-183-0105	EVG/r+FTC/TDF+ZDV	125 mg	TE	B	VF (W48)	Study Drug Not Completed	ND	W48	BL	8720	210	S17N V72I A91E L101I K111T V113I S119R T122I T124A V234L D256E	V72I S119R		102	1.20	1.06	ND	M41L M184V L210W T215Y E138A	G48V I54V V82T L10V F53L I54V I62V L63P A71I G73S V77I I93L
	GS-US-183-0105	EVG/r+FTC/TDF+ZDV	125 mg	TE	B	VF (W48)	Study Drug Not Completed	ND	W48	W8	459	ND	S17N **T66T/A** V72I A91E **T97T/A** L101I K111T V113I S119R T122I T124A V234L D256E	**T66T/A T97T/A** V72I S119R		ND	ND	ND	ND	ND	ND
	GS-US-183-0105	EVG/r+FTC/TDF+ZDV	125 mg	TE	B	VF (W48)	Study Drug Not Completed	ND	W48	W12	776	ND	S17N V72I A91E **T97T/A** L101I K111T V113I S119R T122I T124A **N155N/H K160K/N E170E/A** V234L D256E	**T97T/A N155N/H** V72I S119R **E170E/A**	Maintained	ND	ND	ND	ND	ND	ND
	GS-US-183-0105	EVG/r+FTC/TDF+ZDV	125 mg	TE	B	VF (W48)	Study Drug Not Completed	ND	W48	W16	1400	ND	S17N V72I **A91A/E T97T/A** L101I K111T V113I S119R T122I T124A **N155H K160K/N** V234L D256E	**T97T/A N155H** V72I S119R	Maintained	ND	ND	ND	ND	ND	ND
	GS-US-183-0105	EVG/r+FTC/TDF+ZDV	125 mg	TE	B	VF (W48)	Study Drug Not Completed	ND	W48	W20	1070	ND	S17N V72I **A91A/E** L101I K111T V113I S119R T122I T124A **N155H K160K/N** V234L D256E	**N155H** V72I S119R	Lost	ND	ND	ND	ND	ND	ND
	GS-US-183-0105	EVG/r+FTC/TDF+ZDV	125 mg	TE	B	VF (W48)	Study Drug Not Completed	ND	W48	W24	4960	ND	S17N V72I **A91A/E T97T/A** L101I K111T V113I S119R T122I T124A **N155H E170E/A** V234L D256E	**T97T/A N155H** V72I S119R **E170E/A**	Gained	65	128	21	ND	ND	ND
	GS-US-183-0105	EVG/r+FTC/TDF+ZDV	125 mg	TE	B	VF (W48)	Study Drug Not Completed	ND	W48	ESDD (W40)	6470	ND	S17N V72I A91A/G L101I K111T V113I S119R T122I T124A N155H V234L D256E	**N155H** V72I S119R	Lost	ND	ND	ND	ND	ND	ND
**49**	GS-US-183-0105	EVG/r+ABC+DRV +ENF+FTC/TDF	125 mg	TE	B	VF (W48)	Study Drug Completed	ND	W48	BL	84300	40	E11D R20K S39C V72I L101I V113I S119G T122I T125A I200M V201I T206T/S V234L S255S/T	V72I S119G		95	2.15	1.40	ND	E44E/D T69T/N M184V K103K/N	L33F M46L I54V T74P V82A I84V L90M L10I I13V K20R M36I K43T I54V I62V L63P/T A71V I93L
	GS-US-183-0105	EVG/r+ABC+DRV +ENF+FTC/TDF	125 mg	TE	B	VF (W48)	Study Drug Completed	ND	W48	W4	982	ND	E11D R20K S39C V72I **E92E/Q** L101I V113I S119G T122I T125A I200M V201I V234L	**E92E/Q** V72I S119G		ND	ND	ND	ND	ND	ND
	GS-US-183-0105	EVG/r+ABC+DRV +ENF+FTC/TDF	125 mg	TE	B	VF (W48)	Study Drug Completed	ND	W48	W8	32300	ND	E11D R20K S39C V72I **E92Q** L101I V113I S119G T122I T125A I200M V201I V234L	**E92Q** V72I S119G		ND	ND	ND	ND	ND	ND
	GS-US-183-0105	EVG/r+ABC+DRV +ENF+FTC/TDF	125 mg	TE	B	VF (W48)	Study Drug Completed	ND	W48	W12	9290	ND	E11D R20K S39C V72I **E92Q T97T/A** L101I V113I S119G T122I T125A I200M V201I V234L	**E92Q T97T/A** V72I S119G		ND	ND	ND	ND	ND	ND
	GS-US-183-0105	EVG/r+ABC+DRV +ENF+FTC/TDF	125 mg	TE	B	VF (W48)	Study Drug Completed	ND	W48	W16	3890	ND	E11D R20K S39C V72I **E92Q T97T/A** L101I V113I S119G T122I T125A I200M V201I V234L	**E92Q T97T/A** V72I S119G	Maintained	11	113	27	ND	T69N M184V T215Y	L33F M46L I54V T74P V82A I84V L90M L10I I13V K20R M36I K43T I54V I62V L63P/T A71V I93L
	GS-US-183-0105	EVG/r+ABC+DRV +ENF+FTC/TDF	125 mg	TE	B	VF (W48)	Study Drug Completed	ND	W48	W48	190	ND	ND	ND	Not tested again	ND	ND	ND	ND	ND	ND
	GS-US-183-0130	EVG/r+ABC+DRV +ENF+FTC/TDF	150 mg	TE	B	VF (W48)	Study Drug Completed	ND	-	BL	190	122	ND	ND	Not tested again	ND	ND	ND	ND	ND	ND
	GS-US-183-0130	EVG/r+ABC+DRV +ENF+FTC/TDF	150 mg	TE	B	VF (W48)	Study Drug Completed	ND	-	W32	49	239	ND	ND	Not tested again	ND	ND	ND	ND	ND	ND
**50**	GS-US-183-0145	RAL+ABC+LPV/r	400 mg	TE	B	VF (W96)	Lack Of Efficacy	96%	W48	BL	48100	315	E11D E13D A21T V72I L101I V113I S119G T122I T124S K136N V234L D256E	V72I S119G		ND	1.11	0.88	ND	L210L/W T215Y	L33I I64V
	GS-US-183-0145	RAL+ABC+LPV/r	400 mg	TE	B	VF (W96)	Lack Of Efficacy	96%	W48	W40	911	272	E11D E13D A21T M50M/I V72I T97T/A L101I V113I S119G T122I T124S K136N N155N/H V234L D256E	**T97T/A N155N/H M50M/I** V72I S119G	Gained	ND	3.54	2.44	0.82	T215Y	L33I I64V
	GS-US-183-0145	RAL+ABC+LPV/r	400 mg	TE	B	VF (W96)	Lack Of Efficacy	96%	W48	ESDD (W48)	1250	302	ND	ND	Not tested again	ND	ND	ND	ND	ND	ND
**51**	GS-US-183-0145	RAL+DRV/r+TDF	400 mg	TE	B	VF (W96)	Lack Of Efficacy	95%	W48	BL	177000	43	E11D A23V L28I V37I V72I L101I V113I V201V/I T206S K215N D232D/E V234L D256E S283G	V72I		ND	1.3	1.5	ND	K103N Y181C H221Y	L63P V77I I93L
	GS-US-183-0145	RAL+DRV/r+TDF	400 mg	TE	B	VF (W96)	Lack Of Efficacy	95%	W48	W8	716000	74	E11D A23V L28I V37I V72I L101I V113I V201V/I T206S K215N D232D/E V234L D256E S283G	V72I		ND	1.3	1.2	ND	K103N Y181C H221Y	L63P V77I I93L
	GS-US-183-0145	RAL+DRV/r+TDF	400 mg	TE	B	VF (W96)	Lack Of Efficacy	95%	W48	W20	38800	100	E11D A23V L28I V37I V72I L101I V113I V201V/I T206S K215N D232D/E V234L D256E S283G	V72I		ND	1.4	1.1	ND	K103N Y181C H221Y	L63P V77I I93L
	GS-US-183-0145	RAL+DRV/r+TDF	400 mg	TE	B	VF (W96)	Lack Of Efficacy	95%	W48	W48	24200	83	E11D A23V L28I V37I **D41D/N G70G/R** V72I **T97T/A** L101I V113I **P142P/T V151V/I N155N/H E157E/Q** V201V/I T206S K215N D232D/E V234L D256E S283G	**T97T/A N155N/H** V72I **V151V/I E157E/Q**		ND	21	12.8	ND	**K65K/R K70K/E** K103N Y181C H221Y	**M36M/I** L63P V77I I93L
	GS-US-183-0145	RAL+DRV/r+TDF	400 mg	TE	B	VF (W96)	Lack Of Efficacy	95%	W48	ESDD (W56)	32900	118	E11D A23V L28I V37I **D41D/N** V72I **T97T/A** L101I **T112T/A** V113I **S119S/R V151V/I N155H E157E/Q** V201I T206S K215N D232D/E V234L D256E S283G	**T97T/A N155H** V72I **S119S/R V151V/I E157E/Q**	Maintained	ND	72	57	1.46	**K65R** K103N Y181C H221Y	L63P V77I I93L

PID #	Study #	Treatment Group	INSTI Dose	TE/ TN	HIV-1 Subtype	Snapshot Outcome	Drug Completion Reason	Adhr Rate[a]	RAP[b]	Visit	HIV-1 RNA	CD4	Integrase Sequence[c, d]	INSTI RAM(s)[d]	T97A Status	IN Phenotypic Data[e]				RTI RAM(s)[f]	PI RAM(s)[g]
																RC	EVG FC	RAL FC	DTG FC		
52	GS-US-183-0105	EVG/r+ABC+ddI	150 mg	TE	B	VS (48)	ND	ND	-	BL	49100	597	V72I L101I T112I V113I S119G T122I T124T/A/D/N T125T/A I182V V201V/I Q216H/N T218T/S V234L D256E	V72I S119G		ND	ND	ND	ND	M41L L210W T215Y K103N	L33F M46L I84I/V N88S L90L/M L10I I13V K20R M36I I62V L63L/P I64V A71A/V G73G/S
	GS-US-183-0130	EVG/r+ABC+ddI	150 mg	TE	B	VF (W48)	Study Drug Not Completed	97%	W192	BL	<50	398	V72I L101I T112I V113I S119G T122I T124T/A/D/N T125T/A I182V V201V/I Q216H/N T218T/S V234L D256E	V72I S119G		ND	ND	ND	ND	M41L L210W T215Y K103N	L33F M46L I84I/V N88S L90L/M L10I I13V K20R M36I I62V L63L/P I64V [illegible]
	GS-US-183-0130	EVG/r+ABC+ddI	150 mg	TE	B	VF (W48)	Study Drug Not Completed	97%	W192	W93	268	446	**V31I** K34K/N V72I **T97A** L101I **V113L S119R** T122I T124N **N155H** V201I Q216H T218T/S V234L D256E	**T97A N155H** V72I **S119R**		ND	>140	115	ND	ND	
	GS-US-183-0130	EVG/r+ABC+ddI	150 mg	TE	B	VF (W48)	Study Drug Not Completed	97%	W192	ESDD (W105)	1690	480	**V31I** V72I **T97A** L101I **V113L S119R** T122I T124N **N155H H171H/P** V201I Q216H V234L D256E	**T97A N155H** V72I **S119R**	Maintained	ND	>134	146	ND	M41L **L74L/V V118V/I M184V** L210W T215Y K103N	L33F M46L L10I I13V K20R M36I I62V L63P **I64I/L**/V A71V
53	GS-US-183-0105	EVG/r+ABC +ddI+FTC/TDF	50 mg	TE	B	VS (W48)	ND	ND	-	BL	160000	NA	E11D S24N V72I L101I K111Q V113I S119T T125T/A M154I E212A Q216H V234L	V72I S119T		ND	ND	ND	ND	M41L T215Y E138A	M46I N88D L90M L63P A71V V77I N88D I93L
	GS-US-183-0130	EVG/r+ABC +ddI+FTC/TDF	150 mg	TE	B	VF (W48)	Study Drug Not Completed	100%	W192	BL	52	366	E11D S24N V72I L101I K111Q V113I S119T T125T/A M154I E212A Q216H V234L	V72I S119T		ND	ND	ND	ND	M41L T215Y E138A	M46I N88D L90M L63P A71V V77I N88D I93L
	GS-US-183-0130	EVG/r+ABC +ddI+FTC/TDF	150 mg	TE	B	VF (W48)	Study Drug Not Completed	100%	W192	W84	17000	332	E11D S24N V72I **E92Q T97A** L101I K111Q V113I S119T T125A **S153A** M154I E212A Q216H V234L	**E92Q T97A** V72I S119T **S153A**		ND	>129	28	ND	M41L **M184V** T215Y E138A	M46I N88D L90M L63P A71V V77I N88D I93L
	GS-US-183-0130	EVG/r+ABC +ddI+FTC/TDF	150 mg	TE	B	VF (W48)	Study Drug Not Completed	100%	W192	ESDD (W96)	32300	417	E11D S24N V72I **E92Q T97A** L101I K111Q **T112T/A** V113I S119T **T124T/A** T125A **S153A M154M/I** E212A Q216H V234L	**E92Q T97A** V72I S119T **S153A**	Maintained	ND	>134	18.90	ND	M41L **M184V** T215Y E138A	M46I N88D L90M L63P A71V V77I N88D I93L

[a] Overall adherence rate as determined by pill count in returned pill bottles.

[b] The resistance analysis population (RAP) included subjects with either 1) virologic rebound (VR): HIV-1 RNA ≥50 copies/mL at any visit , that is confirmed and ≥400 copies/mL at the next scheduled or unscheduled visit; or 2) Discontinuation from study with HIV-1 RNA ≥400 copies/mL at last visit (Last) while receiving study drugs (or within 72 hours of discontinuation). Subjects who were not receiving study drugs within 72 hours prior to virologic rebound (Off SD) were not analyzed for resistance.

[c] Monogram Biosciences (South San Francisco, CA) GeneSeq™ Integrase or GenoSure™ Integrase assay

[d] Post-baseline IN sequence was compared to Baseline (BL) or Screening (SCR) IN sequence with emergent RAMs shown in **bold**. Primary INSTI RAMs are: T66A/I/K, E92G/Q, T97A, Y143C/H/R, S147G, Q148H/K/R, and N155H. Secondary INSTI RAMs are: M50I, H51Y, L68I/V, V72A/N/T, L74I/M, Q95K/R, G118R, S119G/P/R/T, F121C/Y, A128T, E138A/K, G140A/C/S, P145S, Q146I/K/L/P/R, V151A/L, S153A/F/Y, E157K/Q, G163K/R, E170A, and R263K. Other variants at IN positions associated with INSTI RAMs are also indicated.

[e] Monogram Biosciences (South San Francisco, CA) PhenoSense™ Integrase assay. Replication capacity (RC) expressed as % of wild-type control. Drug susceptibility data expressed as EC_{50} fold change (FC) from wild-type control.

[f] NRTI mutations are: M41L, E44D, A62V, K65R, D67N, T69D/N, T69 insertion, K70E/R, L74I/V, V75I, F77L, Y115F, F116Y, V118I, Q151M, M184I/V, L210W, T215F/Y, and K219E/N/Q/R in RT. NNRTI-R mutations are: V90I, A98G, L100H/I, K101E/P, K103N/S, V106A/I/M, V108I, E138A/G/K/Q/R, V179D/F/L/T, Y181C/I/V, Y188L/C/H, G190A/E/Q/S, H221Y, P225H, F227C, M230I/L

[g] Primary PI-R mutations are: D30N, V32I, L33F, M46I/L, I47A/V, G48V, I50L/V, I54L/M, Q58E, T74P, L76V, V82A/F/L/S/T, I84V, N88S, and L90M in PR. Secondary PI-R mutations are: L10C/F/I/R/V, V11I, I13V, G16E, K20I/M/R/T/V, L24I, L33I/V, E34Q, E35G, M36I/L/V, K43T, F53L/Y, I54A/S/T/V, D60E, I62V, L63P, I64L/M/V, H69K, A71I/L/T/V, G73A/C/S/T, V77I, V82I, N83D, I85V, N88D, L89V, and I93L/M in PR.

AF: assay failure; DTG: dolutegravir; EVG: elvitegravir; IN: integrase; INSTI: integrase strand transfer inhibitor; ND: no data; PI: protease inhibitor; PID: patient identification; RAL: raltegravir; RAM: resistance-associated mutation; RTI: reverse transcriptase inhibitor; VF: virologic failure; VS: virologic success.
