# Supplementary material for: Lack of impact of pre-existing T97A HIV-1 integrase mutation on integrase strand transfer inhibitor resistance and treatment outcome
Source: PLoS One. 2017 Feb 17;12(2):e0172206. doi: 10.1371/journal.pone.0172206 (PMC5315389; doi:10.1371/journal.pone.0172206)
Supplement: S3 Table — (PDF) [file pone.0172206.s003.pdf]

S3 Table. On-Treatment Population of Patients with Emergent T97A Alone (n = 8)

PID #	Study #	Treatment Group	INSTI Dose	TE/ TN	HIV-1 Subtype	Snapshot Outcome	Drug Completion Reason	Adhr Rate[a]	RAP[b]	Visit	HIV-1 RNA	CD4	Integrase Sequence[c, d]	INSTI RAM(s)[d]	T97A Status	IN Phenotypic Data[e]				RTI RAM(s)[f]	PI RAM(s)[g]
																RC	EVG FC	RAL FC	DTG FC		
**Enrolled on INSTI-based Regimen**																					
54	GS-US-236-0103	EVG/COBI/FTC/TDF	150 mg	TN	B	VF (W144)	Lack Of Efficacy	88%	W144	BL	151000	ND	E11D S17T V37I V72I E96D L101I K111R V113I S119R T125A K156N D167D/E V201I T218T/S V234L D256E	V72I S119R		62	ND	ND	ND	V106I	I62V A71T V77I
	GS-US-236-0103	EVG/COBI/FTC/TDF	150 mg	TN	B	VF (W144)	Lack Of Efficacy	88%	W144	W120	4700	ND	E11D S17T V37I V72I E96D **T97A** L101I K111R V113I S119R T125A K156N D167E V201I **K211R V234F/L** D256E	**T97A** V72I S119R		ND	ND	ND	ND	**M184M/I/V** V106I	I62V A71T V77I
	GS-US-236-0103	EVG/COBI/FTC/TDF	150 mg	TN	B	VF (W144)	Lack Of Efficacy	88%	W144	W132	2480	184	E11D S17T V37I V72I E96D **T97A** L101I K111R V113I S119R T125A K156N **G163G/R** D167E V201I K211K/R V234L D256E	**T97A** V72I S119R **G163G/R**	Maintained	59	ND	ND	ND	**M184V** V106I	I62V A71T V77I
	GS-US-236-0103	EVG/COBI/FTC/TDF	150 mg	TN	B	VF (W144)	Lack Of Efficacy	88%	W144	ESDD (W144)	<50	ND	ND	ND	Not tested again	ND	ND	ND	ND	ND	ND
55	GS-US-183-0145	EVG/r+ETR+LPV	150 mg	TE	B	VS (W96)	Withdrew Consent	99%	-	BL	4260	478	K7R D25E V31I K111T S119P T122I T124N T206S V234I A265V D286N	S119P		ND	ND	ND	ND	M41L T69T/I L74L/I V118I M184V L210W T215Y K219K/E K103N V108V/I	I62V
	GS-US-183-0145	EVG/r+ETR+LPV	150 mg	TE	B	VS (W96)	Withdrew Consent	99%	-	ESDD (W144)	91800	333	**K14K/R** V31I **T97T/A** K111T **V113V/I** S119P T122T/I T124H/N **T125T/A** T206S V234I A265V D286N	**T97T/A** S119P		ND	1.27	0.88	ND		
56	GS-US-183-0145	EVG/r+TDF+DRV	150 mg	TE	B	VF (W96)	Subject Non-Compliance	94%	W96	BL	12200	115	V72I L101I G106G/A V113I T124A K156N V201I I217V V234L P261S R262L	V72I		115	0.78	0.61	ND	K103N	L10I M36I I93L
	GS-US-183-0145	EVG/r+TDF+DRV	150 mg	TE	B	VF (W96)	Subject Non-Compliance	94%	W96	W12	8320	137	V72I **P90P/S** L101I V113I T124A K156N V201I I217V **V234L/F**	V72I		48	0.96	0.50	ND	K103N	L10I M36I I93L
	GS-US-183-0145	EVG/r+TDF+DRV	150 mg	TE	B	VF (W96)	Subject Non-Compliance	94%	W96	W24	567	231	V72I L101I V113I T124A K156N V201I I217V V234L	V72I		133	1.14	0.54	ND	K103N	L10I M36I I93L
	GS-US-183-0145	EVG/r+TDF+DRV	150 mg	TE	B	VF (W96)	Subject Non-Compliance	94%	W96	W56	12800	134	V72I L101I V113I T124A K156N **Q164E** V201I I217V V234L	V72I		ND	1.04	0.46	ND	ND	L10I M36I I93L
	GS-US-183-0145	EVG/r+TDF+DRV	150 mg	TE	B	VF (W96)	Subject Non-Compliance	94%	W96	W72	2410	154	**V31V/M/I V72N/I T97T/A** L101I V113I T124A K156N V201I I217V V234L	**T97T/A V72N/**I		ND	1.93	0.6	ND		L10I M36I I93L
	GS-US-183-0145	EVG/r+TDF+DRV	150 mg	TE	B	VF (W96)	Subject Non-Compliance	94%	W96	W96	53	142	ND	ND	Not tested again	ND	ND	ND	ND	ND	ND
	GS-US-183-0145	EVG/r+TDF+DRV	150 mg	TE	B	VF (W96)	Subject Non-Compliance	94%	W96	ESDD (W112)	4580	134	ND	ND	Not tested again	ND	ND	ND	ND	ND	ND
57	GS-US-183-0145	RAL+ABC+LPV/r	400 mg	TE	B	VF (W96)	Subject Non-Compliance	84%	W144	BL	98100	209	D6E E10D V72V/I A91S V113I S119G T122I T124A T125A I220L Y227F V234I	V72V/I S119G		106	1.44	0.96	ND	D67N	I62V L63P A71T V77I I93L
	GS-US-183-0145	RAL+ABC+LPV/r	400 mg	TE	B	VF (W96)	Subject Non-Compliance	84%	W144	W12	26700	203	D6E E10D V72V/I A91A/S V113I S119G T122I T124A T125A I220L Y227F V234I	V72V/I S119G		ND	1.32	0.89	ND	D67N	I62V L63P A71T V77I I93L
	GS-US-183-0145	RAL+ABC+LPV/r	400 mg	TE	B	VF (W96)	Subject Non-Compliance	84%	W144	W104	99800	251	D6E E10D **L74L/M** A91S **T97A K103K/R** V113I S119G T122I T124A T125A **V151V/I** I220L Y227F V234I	**T97A L74L/M** S119G **V151V/I**		58	8.95	2.19	0.57		
	GS-US-183-0145	RAL+ABC+LPV/r	400 mg	TE	B	VF (W96)	Subject Non-Compliance	84%	W144	ESDD (W112)	68500	357	ND	ND	Not tested again	ND	ND	ND	ND	ND	ND
58	GS-US-183-0145	RAL+ABC+LPV/r	400 mg	TE	B	VF (W96)	Lack Of Efficacy	92%	W48	BL	22400	381	E10D R20R/K M50I V72I L101I T112I V113I S119P T124A T125A I135L T206S V234L D279D/N	M50I V72I S119P		65	1.27	0.74	ND	D67N K70R K103N P225H	V32I L90M L10I K20R M36I K43T I64V G73C I85V
	GS-US-183-0145	RAL+ABC+LPV/r	400 mg	TE	B	VF (W96)	Lack Of Efficacy	92%	W48	W24	5320	440	**D6D/N** E10D **S39S/G** M50I V72I **L74M T97A** L101I T112I V113I S119P T124A T125A I135L T206S V234L D279N	**T97A** M50I V72I **L74M** S119P		49	9.94	2.61	0.55	D67D/N K70K/R K103K/N **V179V/D** P225P/H	V32V/I L90L/M L10L/I K20K/R M36M/I K43K/T I64I/V G73G/C I85I/V
	GS-US-183-0145	RAL+ABC+LPV/r	400 mg	TE	B	VF (W96)	Lack Of Efficacy	92%	W48	ESDD (W40)	51000	384	E10D M50I V72I **L101I/V** T112I V113I S119P T124A T125A **K136K/E G163G/R** T206S V234L **R269R/K** D279D/N **D288D/N**	M50I V72I S119P **G163G/R**	Lost	ND	1.50	1.09	ND	K103K/N **V179V/I**	K43K/T I64I/V
59	GS-US-183-0145	EVG/r+MVC+DRV	150 mg	TE	B	VF (W96)	Study Drug Completed	97%	W48	BL	82800	157	K14R R20R/K V72I A91E L101I V113I S119R T124N G193D V201I T206S I208I/L V234L D256D/E I268L R269R/K D270D/H	V72I S119R		177	1.21	0.97	ND	L74I M184V K103N G190A	M36I L63P
	GS-US-183-0145	EVG/r+MVC+DRV	150 mg	TE	B	VF (W96)	Study Drug Completed	97%	W48	W40	1180	332	K14R R20R/K V72I A91E **T97A** L101I V113I S119R T124N G193D V201I T206S I208I/L V234L I268L **D279G**	**T97A** V72I S119R		66	5.37	2.18	ND	K103N	**L10L/F** M36M/I L63P **I93I/L**
	GS-US-183-0145	EVG/r+MVC+DRV	150 mg	TE	B	VF (W96)	Study Drug Completed	97%	W48	W48	1060	321	K14R R20R/K V72I A91E **T97A** L101I V113I S119R T124N G193D V201I T206S I208I/L V234L I268L **D279G**	**T97A V72I** S119R	Maintained	ND	4.64	1.93	ND	K103N	L63P **I93I/L**
	GS-US-183-0145	EVG/r+MVC+DRV	150 mg	TE	B	VF (W96)	Study Drug Completed	97%	W48	W96	595	453	K14R V72I A91E **T97A** L101I **T112A** V113I S119R T124N G193D V201I T206S I208L **V234L/F** I268L R269R/K **D279G**	**T97A** V72I S119R	Maintained	6.3	6.62	2.99	0.7	ND	ND
	GS-US-183-0145	EVG/r+MVC+DRV	150 mg	TE	B	VF (W96)	Study Drug Completed	97%	W48	W144	410	461	ND	ND	Not tested again	ND	ND	ND	ND	ND	ND
	GS-US-183-0145	EVG/r+MVC+DRV	150 mg	TE	B	VF (W96)	Study Drug Completed	97%	W48	W144 OL	305	751	ND	ND	Not tested again	ND	ND	ND	ND	ND	ND

PID #	Study #	Treatment Group	INSTI Dose	TE/ TN	HIV-1 Subtype	Snapshot Outcome	Drug Completion Reason	Adhr Rate[a]	RAP[b]	Visit	HIV-1 RNA	CD4	Integrase Sequence[c, d]	INSTI RAM(s)[d]	T97A Status	IN Phenotypic Data[e]				RTI RAM(s)[f]	PI RAM(s)[g]
																RC	EVG FC	RAL FC	DTG FC		
60	GS-US-183-0145	EVG/r+TDF+DRV	150 mg	TE	B	VF (W96)	Lack Of Efficacy	96%	W48	BL	30400	287	S39C M50I V72I L101I V113I S119P M154L K173K/R Q177Q/L V201I T206S T218S V234L	M50I V72I S119P		106	1.31	1.13	ND	V90V/I	I93I/L
	GS-US-183-0145	EVG/r+TDF+DRV	150 mg	TE	B	VF (W96)	Lack Of Efficacy	96%	W48	W20	5700	263	S39C M50I V72I L101I V113I S119P **T125T/A** M154L V201I T206S T218S V234L **S255S/G**	M50I V72I S119P		133	2.13	0.86	ND	V90V/I	I93I/L
	GS-US-183-0145	EVG/r+TDF+DRV	150 mg	TE	B	VF (W96)	Lack Of Efficacy	96%	W48	W48	22400	284	S39C M50I V72I **V77V/A T97T/A** L101I V113I S119P M154L V201I T206S T218S V234L	**T97T/A** M50I V72I S119P		ND	3.92	1.05	ND	**K219K/R** V90V/I	
	GS-US-183-0145	EVG/r+TDF+DRV	150 mg	TE	B	VF (W96)	Lack Of Efficacy	96%	W48	ESDD (W56)	27700	292	S39C M50I V72I **T97A** L101I V113I S119P M154L V201I T206S T218S V234L	**T97A** M50I V72I S119P	Maintained	ND	6.64	1.65	0.7	**K219K/R** V90V/I	
61	GS-US-183-0145	EVG/r+FTC/TDF+LPV	150 mg	TE	B	VF (W96)	Subject Non-Compliance	78%	W48	SCR	570	567	S17N L28I V32V/I S39C M50M/I V113I E138E/K V201I K211Q V234L	M50M/I E138E/K		170	1.21	0.91	ND	M41L D67N K70R M184M/V T215Y K219E E138A	I54L L10I I13I/V G16E D60E L63P I93L
	GS-US-183-0145	EVG/r+FTC/TDF+LPV	150 mg	TE	B	VF (W96)	Subject Non-Compliance	78%	W48	W12	773	506	S17N L28I S39C M50M/I **T97T/A** V113I **K156K/R** V201I K211Q V234L **S283S/G**	**T97T/A** M50M/I		62	1.56	0.79	ND	M41L D67N K70R M184M/V T215Y K219E E138A	I54L L10I I13I/V G16E D60E L63P **I85I/V** I93L
	GS-US-183-0145	EVG/r+FTC/TDF+LPV	150 mg	TE	B	VF (W96)	Subject Non-Compliance	78%	W48	ESDD (W24)	1330	557	S17N L28I S39C M50M/I **T66T/A** V113I **T124T/A S147S/G** V201I K211Q V234L	**T66T/A S147S/G** M50M/I	Switched INSTI-R pathway(s)	ND	5.00	1.05	0.73	ND	ND

[a] Overall adherence rate as determined by pill count in returned pill bottles.

[b] The resistance analysis population (RAP) included subjects with either 1) virologic rebound (VR): HIV-1 RNA ≥50 copies/mL at any visit , that is confirmed and ≥400 copies/mL at the next scheduled or unscheduled visit; or 2) Discontinuation from study with HIV-1 RNA ≥400 copies/mL at last visit (Last) while receiving study drugs (or within 72 hours of discontinuation). Subjects who were not receiving study drugs within 72 hours prior to virologic rebound (Off SD) were not analyzed for resistance.

[c] Monogram Biosciences (South San Francisco, CA) GeneSeq™ Integrase or GenoSure™ Integrase assay

[d] Post-baseline IN sequence was compared to Baseline (BL) or Screening (SCR) IN sequence with emergent RAMs shown in **bold**. Primary INSTI RAMs are: T66A/I/K, E92G/Q, T97A, Y143C/H/R, S147G, Q148H/K/R, and N155H. Secondary INSTI RAMs are: M50I, H51Y, L68I/V, V72A/N/T, L74I/M, Q95K/R, T97A, G118R, S119G/P/R/T, F121C/Y, A128T, E138A/K, G140A/C/S, P145S, Q146I/K/L/P/R, V151A/L, S153A/F/Y, E157K/Q, G163K/R, E170A, and R263K. Other variants at IN positions associated with INSTI RAMs are also indicated.

[e] Monogram Biosciences (South San Francisco, CA) PhenoSense™ Integrase assay. Replication capacity (RC) expressed as % of wild-type control. Drug susceptibility data expressed as EC_{50} fold change (FC) from wild-type control.

[f] NRTI mutations are: M41L, E44D, A62V, K65R, D67N, T69D/N, T69 insertion, K70E/R, L74I/V, V75I, F77L, Y115F, F116Y, V118I, Q151M, M184I/V, L210W, T215F/Y, and K219E/N/Q/R in RT. NNRTI-R mutations are: V90I, A98G, L100H/I, K101E/P, K103N/S, V106A/I/M, V108I, E138A/G/K/Q/R, V179D/F/L/T, Y181C/I/V, Y188L/C/H, G190A/E/Q/S, H221Y, P225H, F227C, M230I/L

[g] Primary PI-R mutations are: D30N, V32I, L33F, M46I/L, I47A/V, G48V, I50L/V, I54L/M, Q58E, T74P, L76V, V82A/F/L/S/T, I84V, N88S, and L90M in PR. Secondary PI-R mutations are: L10C/F/I/R/V, V11I, I13V, G16E, K20I/M/R/T/V, L24I, L33I/V, E34Q, E35G, M36I/L/V, K43T, F53L/Y, I54A/S/T/V, D60E, I62V, L63P, I64L/M/V, H69K, A71I/L/T/V, G73A/C/S/T, V77I, V82I, N83D, I85V, N88D, L89V, and I93L/M in PR.

AF: assay failure; DTG: dolutegravir; EVG: elvitegravir; IN: integrase; INSTI: integrase strand transfer inhibitor; ND: no data; PI: protease inhibitor; PID: patient identification; RAL: raltegravir; RAM: resistance-associated mutation; RTI: reverse transcriptase inhibitor; VF: virologic failure; VS: virologic success.
