# Supplementary material for: Lack of impact of pre-existing T97A HIV-1 integrase mutation on integrase strand transfer inhibitor resistance and treatment outcome
Source: PLoS One. 2017 Feb 17;12(2):e0172206. doi: 10.1371/journal.pone.0172206 (PMC5315389; doi:10.1371/journal.pone.0172206)

## S1 Fig. Pre-Treatment Population of Patients with Pre-Existing T97A (n = 47): Longitudinal Plots

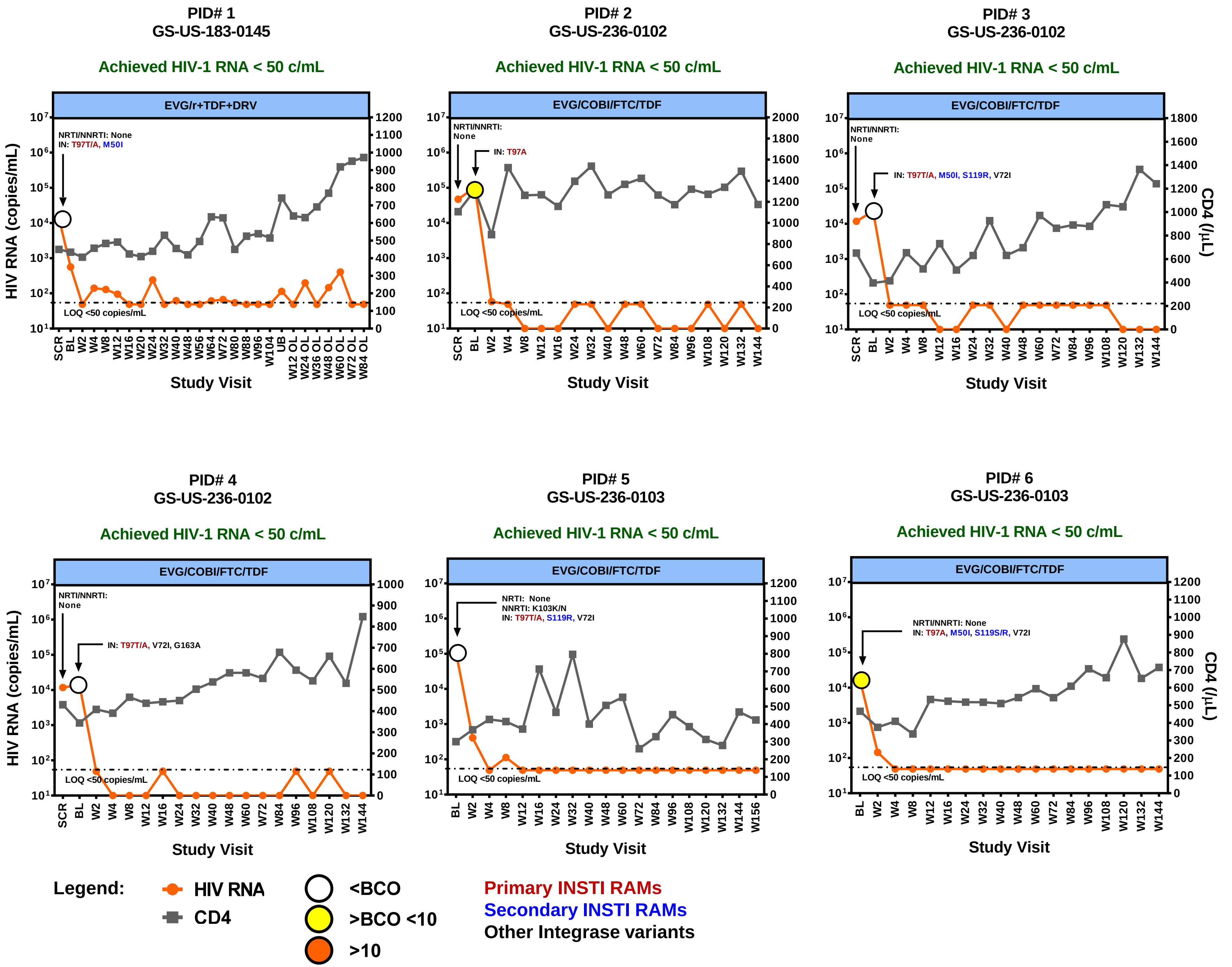

## S1 Fig. Pre-Treatment Population of Patients with Pre-Existing T97A (n = 47): Longitudinal Plots (con't)

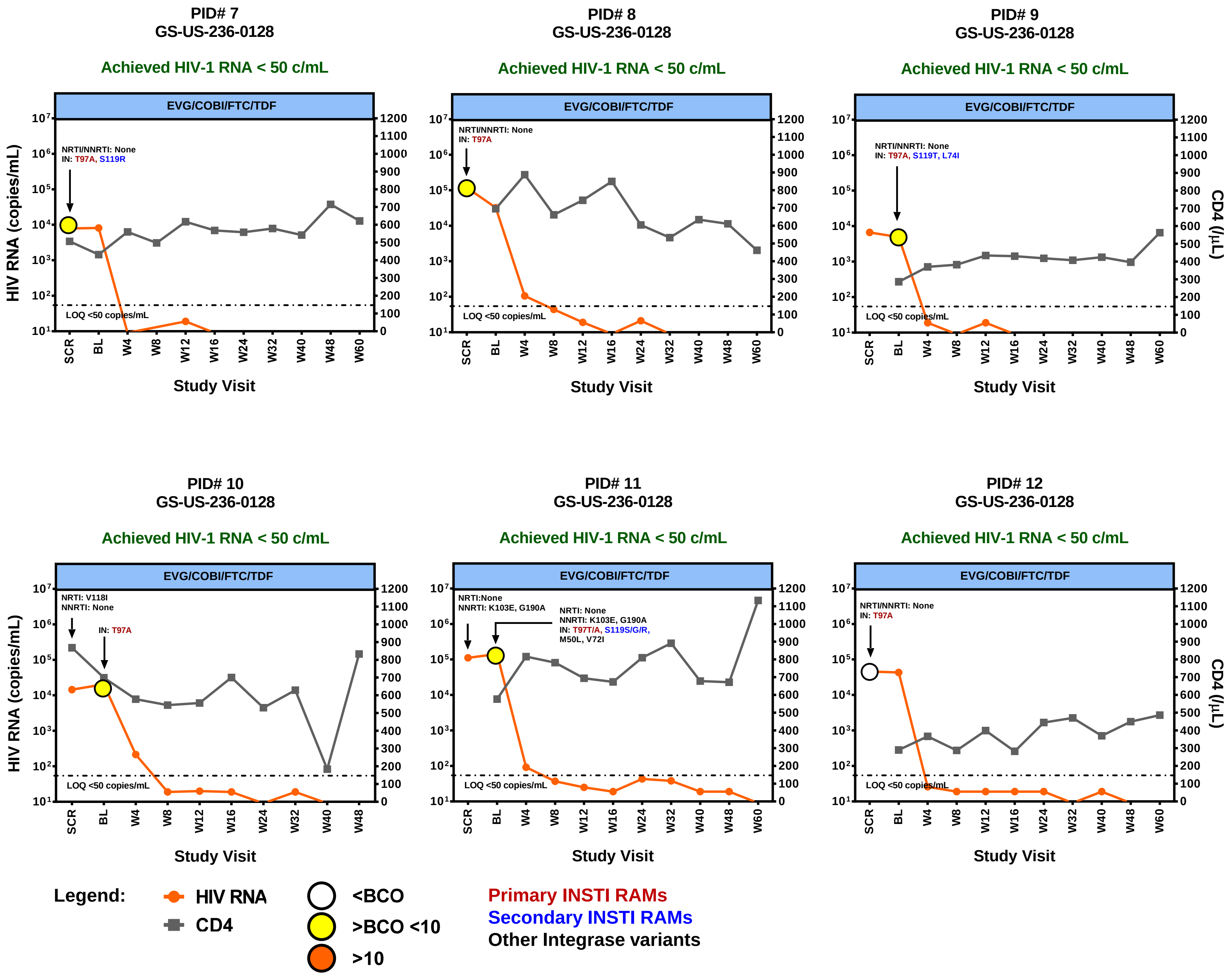

**S1 Fig. Pre-Treatment Population of Patients with Pre-Existing T97A (n = 47): Longitudinal Plots (con't)**

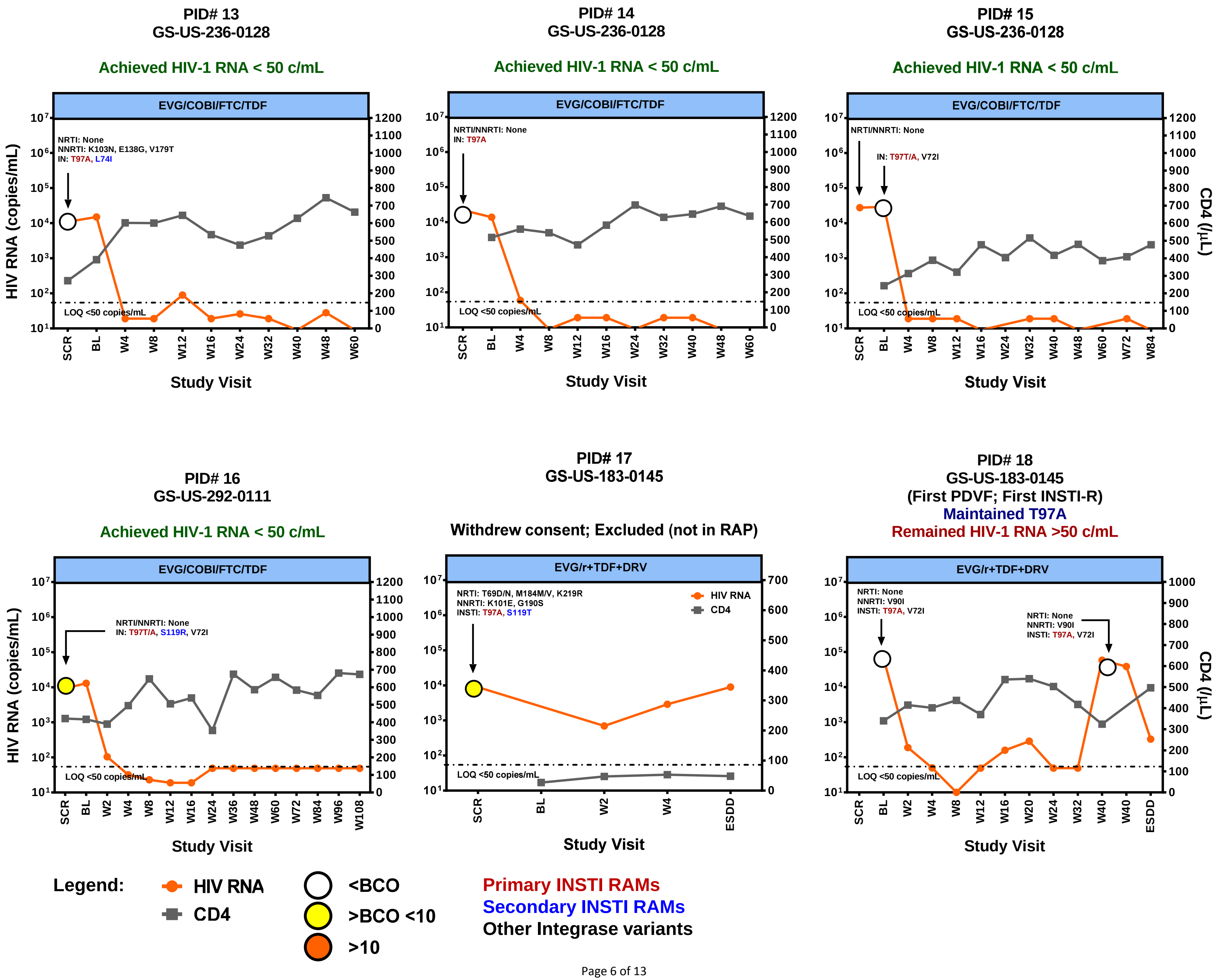

Supplement: S1 Fig — (PDF) [file pone.0172206.s004.pdf]
