# Supplementary material for: Lack of impact of pre-existing T97A HIV-1 integrase mutation on integrase strand transfer inhibitor resistance and treatment outcome
Source: PLoS One. 2017 Feb 17;12(2):e0172206. doi: 10.1371/journal.pone.0172206 (PMC5315389; doi:10.1371/journal.pone.0172206)

S2 Fig. On-Treatment Population of Patients with Emergent T97A and Primary INSTI RAM(s) (n = 6): Longitudinal Plots

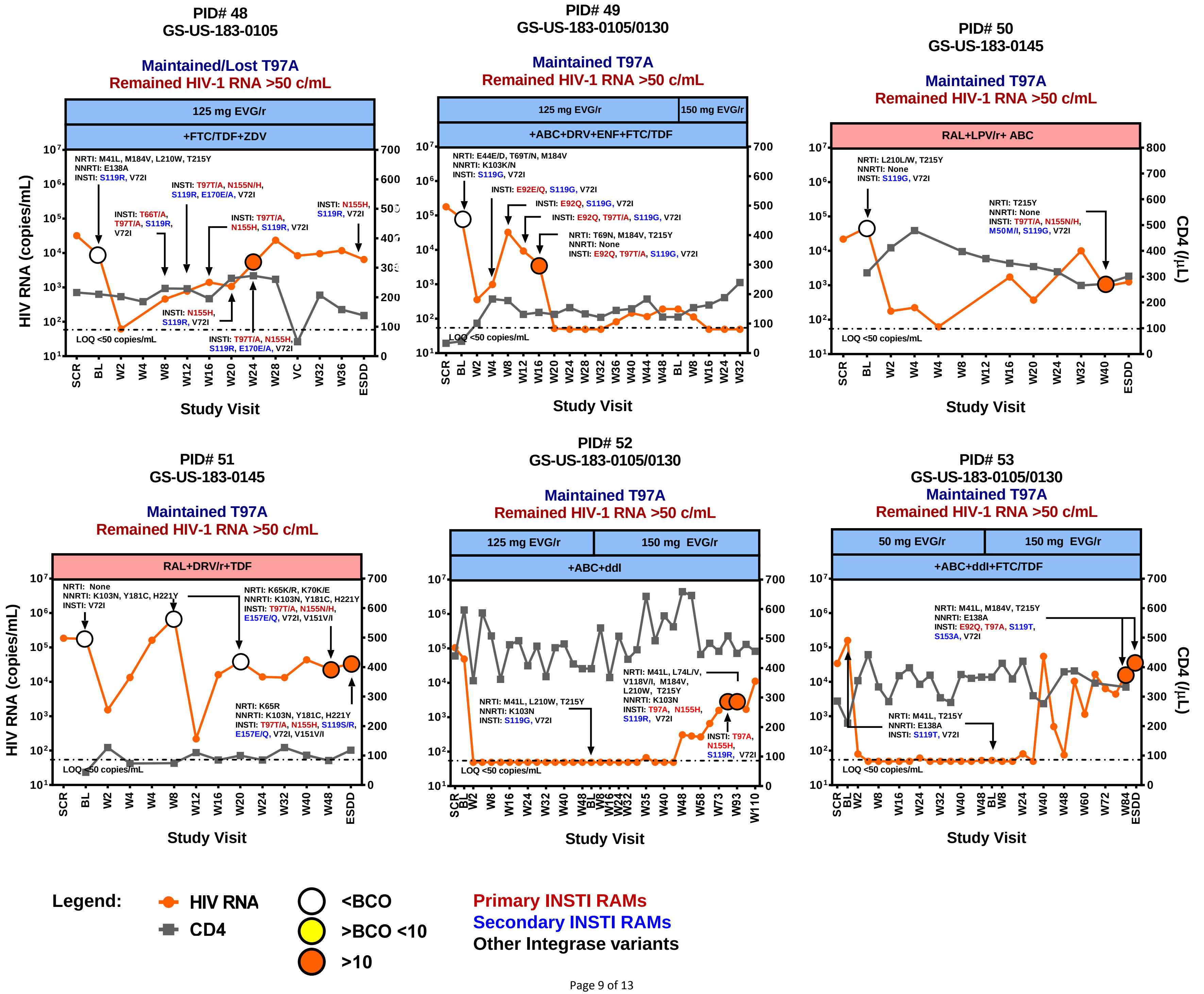

Supplement: S2 Fig — (PDF) [file pone.0172206.s005.pdf]
