# Supplementary material for: Lack of impact of pre-existing T97A HIV-1 integrase mutation on integrase strand transfer inhibitor resistance and treatment outcome
Source: PLoS One. 2017 Feb 17;12(2):e0172206. doi: 10.1371/journal.pone.0172206 (PMC5315389; doi:10.1371/journal.pone.0172206)
Supplement: S3 Fig — (PDF) [file pone.0172206.s006.pdf]

S3 Fig. On-Treatment Population of Patients with Emergent T97A Alone (n = 8): Longitudinal Plots

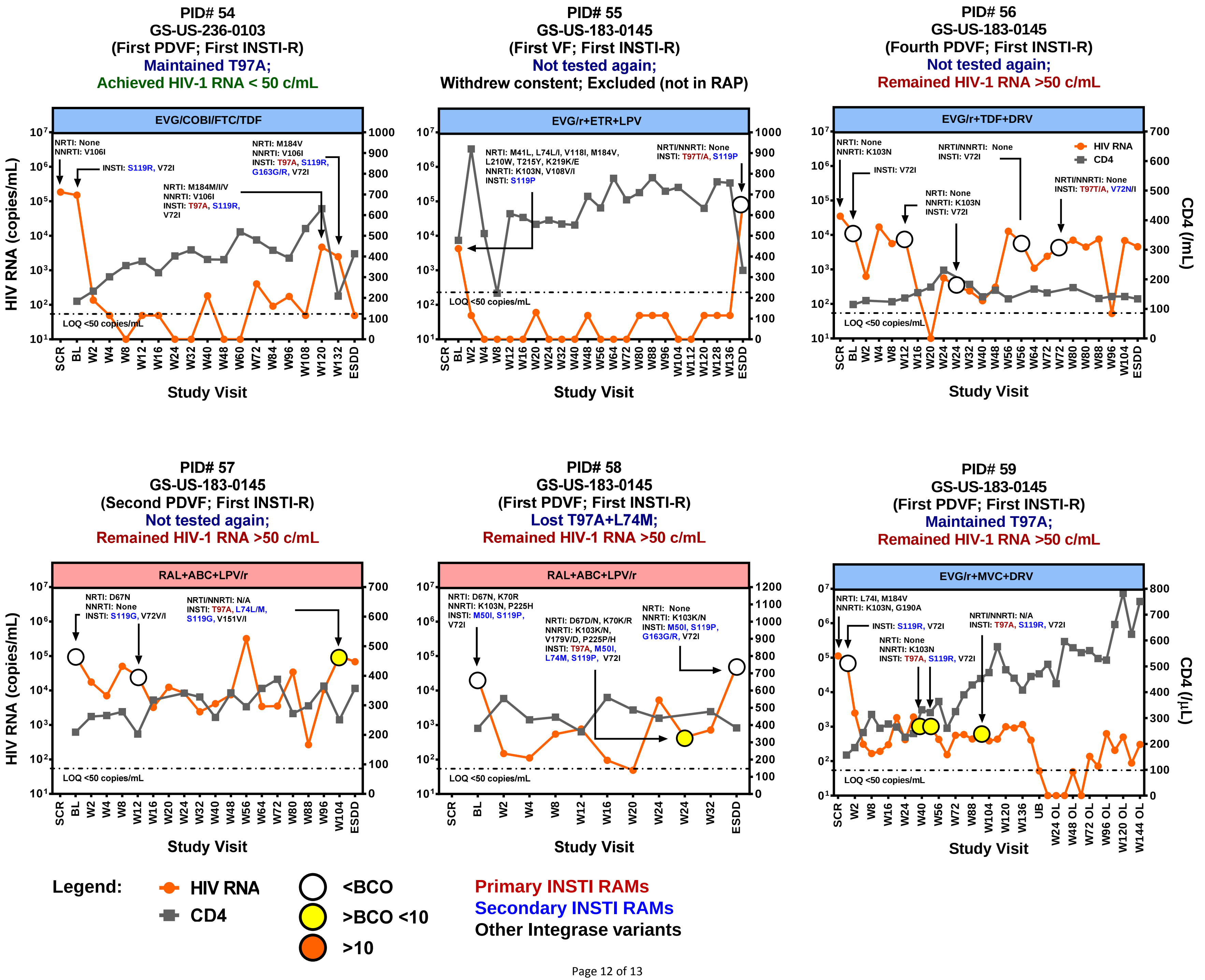

S3 Fig. On-Treatment Population of Patients with Emergent T97A Alone (n = 8): Longitudinal Plots (con’t)

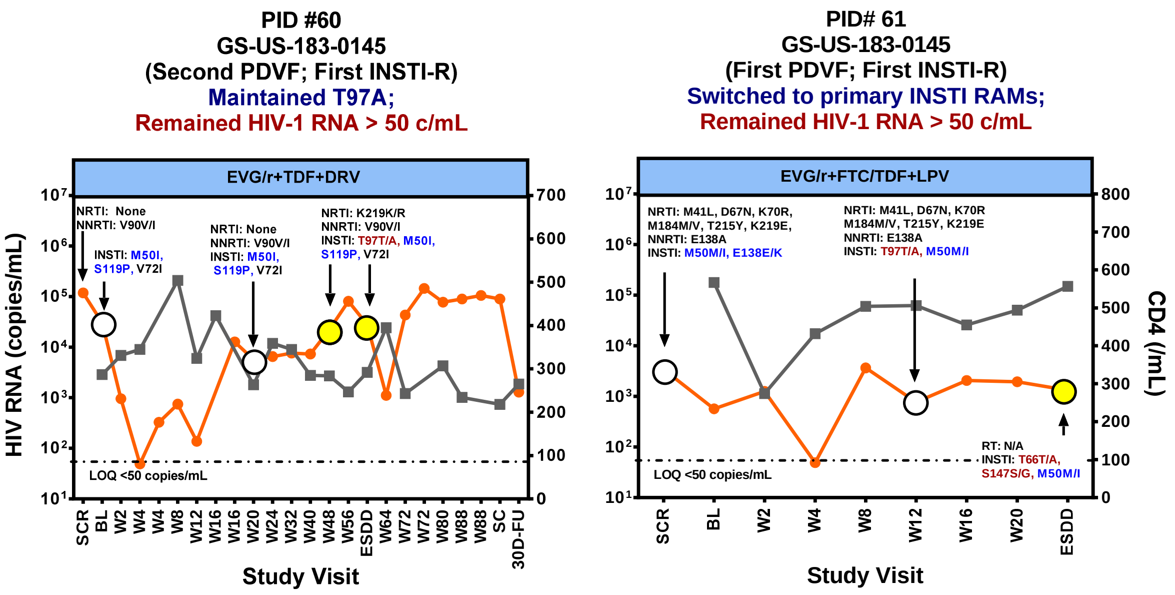

Legend:

- HIV RNA
- CD4
- <BCO
- >BCO <10
- >10

Primary INSTI RAMs
Secondary INSTI RAMs
Other Integrase variants
